# Global longitudinal active strain energy density (GLASED): a powerful prognostic marker in a community-based cohort

**DOI:** 10.1093/ehjci/jeae133

**Published:** 2024-05-20

**Authors:** Nay Aung, David H MacIver, Henggui Zhang, Sucharitha Chadalavada, Steffen E Petersen

**Affiliations:** William Harvey Research Institute, NIHR Barts Biomedical Research Centre, Queen Mary University London, Charterhouse Square, London EC1M 6BQ, UK; Barts Heart Centre, St Bartholomew’s Hospital, Barts Health NHS Trust, West Smithfield, London EC1A 7BE, UK; Department of Astronomy and Physics, Biological Physics Group, University of Manchester, Manchester M13 9PL, UK; Department of Cardiology, Taunton and Somerset Hospital, Somerset TA1 5DA, UK; Department of Astronomy and Physics, Biological Physics Group, University of Manchester, Manchester M13 9PL, UK; William Harvey Research Institute, NIHR Barts Biomedical Research Centre, Queen Mary University London, Charterhouse Square, London EC1M 6BQ, UK; Barts Heart Centre, St Bartholomew’s Hospital, Barts Health NHS Trust, West Smithfield, London EC1A 7BE, UK; William Harvey Research Institute, NIHR Barts Biomedical Research Centre, Queen Mary University London, Charterhouse Square, London EC1M 6BQ, UK; Barts Heart Centre, St Bartholomew’s Hospital, Barts Health NHS Trust, West Smithfield, London EC1A 7BE, UK

**Keywords:** contractance, contractility, contractile function, energy, heart failure, systolic function, stroke work

## Abstract

**Aims:**

Identifying the imaging method that best predicts all-cause mortality, cardiovascular adverse events, and heart failure risk is crucial for tailoring optimal management. Potential prognostic markers include left ventricular (LV) myocardial mass, ejection fraction, myocardial strain, stroke work, contraction fraction, pressure–strain product, and a new measurement called global longitudinal active strain density (GLASED). This study sought to compare the utility of 23 potential LV prognostic markers of structure and contractile function in a community-based cohort.

**Methods and results:**

The impact of cardiovascular magnetic resonance image–derived markers extracted by machine learning algorithms was compared with the future risk of adverse events in a group of 44 957 UK Biobank participants. Most markers, including the LV ejection fraction, have limited prognostic value. GLASED was significantly associated with all-cause mortality and major adverse cardiovascular events, with the largest hazard ratio, highest ranking, and differentiated risk in all three tertiles (*P ≤* 0.0003).

**Conclusion:**

GLASED predicted all-cause mortality and major cardiovascular adverse events better than conventional markers of risk and is recommended for assessing patient prognosis.

## Graphical Abstract

**Graphical Abstract:**
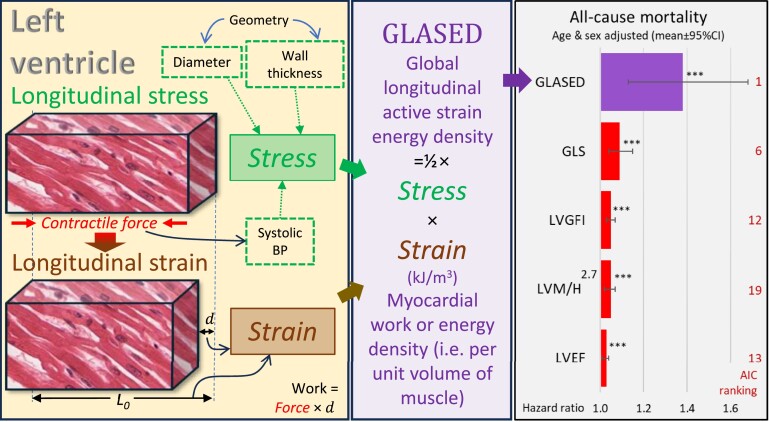

## Introduction

Determining the optimal predictive imaging markers in patients with left ventricular (LV) heart disease is crucial for guiding management decisions. However, the most reliable measures of LV structure or function for determining future risk are unclear.

In this study, we compared the predictive performance of the recognized measures of LV structure and function, with a particular focus on a new method called global longitudinal active strain energy density (GLASED).^1^

At least 23 LV imaging markers, including LV ejection fraction (LVEF),^2^ end-diastolic volume,^3^ LV mass,^4^ myocardial strain,^5^ strain rate,^6^ pressure–strain product,^7^ stroke work, stroke work indexed to LV mass,^1^ global function index,^8^ contraction fraction,^9^ and GLASED, have been advocated for the assessment of contractile function and/or risk.^1^ However, to date, a comprehensive assessment of the relative effectiveness of these potential risk markers has not been undertaken.

Although the LVEF is widely used for risk assessment, it has serious limitations in predicting the prognosis of patients with heart failure (HF) syndromes.^2,10–12^ While myocardial strain has some prognostic value,^6,13^ it is limited by its inability to consider afterload.^5^ A greater afterload results in reduced myocardial shortening.^14,15^ Therefore, afterload not only impacts strain interpretation but also indirectly affects the LVEF because the LVEF itself is influenced by strain.^16^ An accurate method to correct myocardial shortening for afterload has been sought for more than two decades.^15^

Contractance is a new measure of contractile function derived from the area under a stress–strain curve^14^ and can be estimated in whole ventricles using GLASED.^1^ GLASED overcomes the limitations of other methods, including strain, by accurately allowing for the effects of afterload, including remodelling. GLASED estimates the mechanical energy (work done) per unit volume of myocardium during contraction. As such, it is likely to be a more robust measure of myocardial contractile function than alternative measures. Blood pressure (BP), wall thickness, chamber dimensions (determinants of wall stress), and myocardial strain are required for its calculation. GLASED confers a robust theoretical advantage over other approaches for evaluating LV systolic function because strain energy density has a strong background in engineering science. Furthermore, a recent study of a large cohort of patients referred for cardiovascular magnetic resonance (CMR) imaging showed that GLASED is the best predictor of expected prognosis and B-type natriuretic peptide (BNP) compared with the LVEF, stroke work, stroke work per LV mass, pressure–strain product, LV contraction fraction (LVCF), and strain.^1^ Moreover, according to echocardiographic analyses, the GLASED was significantly lower in veteran athletes than in young athletes and greater in young male athletes than in young female athletes.^17^

Global longitudinal active strain energy (GLASE), calculated by multiplying GLASED with the LV muscle volume (LVMV), is a measure of the total work performed by the LV muscle in the longitudinal direction. GLASE provides similar information to stroke work but is derived from information obtained from the myocardium rather than from the lumen. Therefore, GLASE accounts for myocardial shortening, geometric differences (wall thickness and internal dimensions), systolic BP, and muscle volume, whereas stroke work accounts for only systolic pressure and stroke volume. While GLASED measures the myocardium’s health or (dys)function, GLASE indicates whether there is sufficient myocardium (adequate hypertrophy) to generate the energy necessary for a normal LV output that meets the needs of the body.

Given the theoretical benefits of GLASED, combined with the recent prognostic CMR study,^1^ our principal *a priori* hypothesis was that GLASED would be a superior predictor of outcome compared with more conventional markers. Therefore, we aimed to evaluate the impact of various measures of LV structure and contractile function on all-cause mortality (primary endpoint) with major adverse cardiovascular events (MACEs) and HF risk (secondary endpoints) in a community-based longitudinal cohort study using the UK Biobank database.

## Methods

### Study cohort

The UK Biobank is a prospectively recruited population study of more than 500 000 volunteers living in the UK that provides information on demographics, lifestyle, medical background, physical measurements, genomics, and proteomics.^18^ The UK Biobank population cohort was 40–69 years of age and was initially recruited between 2006 and 2010. The enrichment of the original cohort with imaging data, including CMR data, commenced in 2014. The UK Biobank aimed to recruit a cohort that is representative of the UK population sample. Consequently, the investigators did not seek healthy volunteers or oversample people with any health conditions. Ethical approval for the UK Biobank studies was obtained from the NHS National Research Ethics Service on 17 June 2011 (ref.: 11/NW/0382); this approval was extended on 18 June 2021 (ref.: 21/NW/0157).

### Imaging analysis

In-depth information on the UK Biobank CMR imaging protocol commencing in 2014 is available elsewhere.^19^ In total, CMR images from 44 957 individuals were available at the time of this study. Segmentation and derivation of other LV markers were performed using a fully convolutional neural network trained on expert-annotated data from the first 4875 CMR studies, as previously described.^20,21^ We excluded individuals with inadequate quality imaging data as detailed in prior publications.^22,23^

### LV assessment

Peak global longitudinal strain (GLS) was measured using a feature-tracking algorithm implemented in CVI42 software (Circle, prototype v5.13.7). The GLS was calculated using all three long-axis images, i.e. three-chamber, two-chamber, and four-chamber views. Endo- and epicardial contours (excluding extreme basal slices that included the LV outflow tract) were drawn using automated algorithms. Feature tracking markers were attached to the whole myocardium within the defined epicardial and endocardial borders and tracked throughout the cardiac cycle with end diastole as the reference phase. The calculation was made from the longitudinal change detected by multiple trackers of the myocardium to produce the peak GLS using the Green–Lagrange strain method. The LVMV was measured using rounded contours at the blood/myocardial border and excluded papillary muscles and trabeculations. The LV mass was calculated from the LVMV multiplied by the density of myocardial tissue (1.05 g/mL). The LV mass was indexed to body surface area (BSA) and height^2.7^ (height in metres to a power of 2.7).

The LV global function index (LVGFI) was calculated from the following equation^8^:


LVGFI=SV/[LVMV+(ESV+EDV)/2]

where SV is the stroke volume, ESV is the end-systolic volume, and EDV is the end-diastolic volume.

The LVCF (myocardial) was calculated as follows^9^:


LVCF=SV/LVMV

Stroke work was estimated from the product of stroke volume and systolic BP.^1^ Stroke work was indexed to the BSA and height^2.7^.

The pressure–strain product (%mmHg) was calculated as the product of the absolute strain (%) and the systolic BP (mmHg).^7^

The Lamé equation for longitudinal stress is more accurate than other methods and matches the results of LV finite-element analysis.^24^ The peak nominal Lamé longitudinal stress ($\sigma_{l})$ was calculated using the following equation^1,24^:


$$\sigma_{l}=\frac{P_{i}r_{i}^{2}}{r_{o}^{2}-r_{i}^{2}}$$

where *P_i_* is the inner pressure (in Pa) and is equal to the peak systolic pressure measured using a brachial cuff. Furthermore, *r_o_* is the outer (epicardial) radius, and *r_i_* is the inner (luminal or endocardial) LV radius at end-diastole. The inner luminal diameter was calculated as an average value of the measurements from the LV endocardial contours of the basal-to-mid short-axis slices. The end-diastolic maximum wall thickness, which is required to estimate the outer (epicardial) radius, was calculated by taking the mean value of the wall thickness measurements in the American Heart Association segments of the same basal-to-mid short-axis slices. The nominal (i.e. based on the pre-deformed configuration), rather than the instantaneous, stress was used because it correlates well with the numerical calculation (i.e. contractance).^1,17^

GLASED was calculated using the following equation^1^:


$$\mathrm{GLASED}=\frac{1}{2}\times |\sigma_{l}|\times |\varepsilon_{l}|\;\left(i.e.\;\frac{1}{2}\times \mathrm{longitudinal}\;\mathrm{stress}\times \mathrm{peak}\;\mathrm{GLS}\right)$$

where $\left|\varepsilon_{l}\right|$ and $|\sigma_{l}|\;$ are the absolute value (magnitude) of the nominal stress and peak GLS derived by tissue tracking, respectively, to yield a positive value for the GLASED.

GLASE was calculated using the following equation:^1^


GLASE=GLASED×LVMV

GLASE was indexed to both BSA and height^2.7^.

### Prognosis

The primary endpoint was all-cause mortality, and the secondary endpoints were MACEs and HF. Longitudinal follow-up was performed via linkage to Hospital Episodes Statistics data encoded in the International Classification of Disease 10th Revision (ICD10) classification system and national death registries. The ICD10 codes used to define MACE (which includes nonfatal or fatal myocardial infarction and stroke) and HF are detailed in Supplementary data online, *Table S1*.

### Statistical analysis

The full details of the statistical analyses are summarized in Supplementary data. In brief, four main analyses were performed: (i) Cox proportional hazard ratios (HRs) adjusted for age and sex (Model 1) and age and sex with all cardiovascular risk factors (Model 2); (ii) The Akaike information criterion (AIC) was used to rank the predictive accuracy of the markers in Models 1 and 2; (iii) Kaplan–Meier survival analysis showing the unadjusted associations between LV markers and outcomes; and (iv) *C*-statistics. A further subgroup analysis was performed on individuals with an LVEF > 55%. A Holm–Bonferroni-corrected *P*-value of <0.05 was considered to indicate statistical significance.

## Results

### Demographics

The baseline characteristics of the study population are presented in Supplementary data online, *Table S2A*. Our cohort consisted of 21 631 males and 23 326 females, and the overall mean age [standard deviation (SD)] was 64 (8) years. The mean, SD, median, minimum, and maximum values for the LV markers are also shown (see Supplementary data online, *Table S2A*), and their distributions are shown in Supplementary data online, *Figure S1*. The participant characteristics stratified by cardiovascular disease (CVD) status are presented in Supplementary data online, *Table S2B*. The maximum follow-up duration was 6.8 years.

### Relationships between LV markers and age, sex, and risk factors

Age was associated with a smaller LV end-diastolic diameter and lower volume, and lower LVCF, LVGFI, absolute GLS, and GLASED (see Supplementary data online, *Figure S2*). With increasing age, a higher wall stress, pressure–strain product, and stroke work were observed.

The LV mass, the end-diastolic diameter, and volume were lower in females than in males. The LVEF, LVGFI, LVCF, and absolute GLS and GLASED were significantly greater in females than in males (*P* < 0.0001). The presence of the cardiovascular risk factors smoking, alcohol consumption, hypertension, diabetes mellitus, and hyperlipidaemia were consistently associated with poorer LV functional markers such as the LVEF, LVCF, LVGFI, absolute GLS, and GLASED.

A higher physical activity score was associated with a lower LVEF and greater LV dimensions, volumes, mass, LVCF, absolute GLS, and GLASED, but there was no difference in the LVGFI (see Supplementary data online, *Figure S2*).

### Prognostic association with all-cause mortality

**Figure 1:**
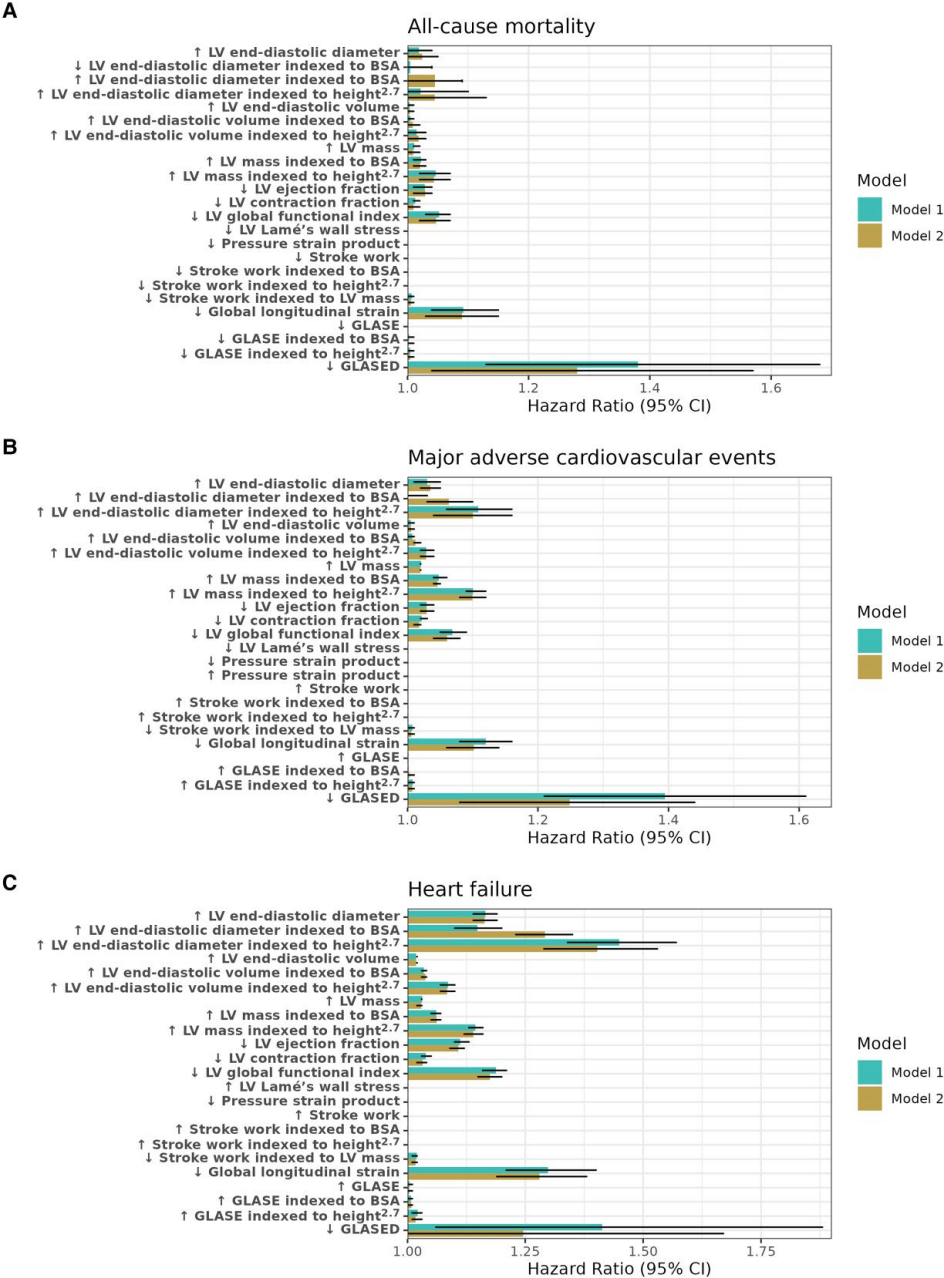
Cox proportional HRs for potential prognostic markers corrected for age and sex (Model 1) and all risk factors (Model 2). The HRs and 95% CIs represent the prognostic association of outcomes with 1 SD change (either increase or decrease, as indicated by the arrow) in LV markers. Model 1 was adjusted for age and sex, and Model 2 was adjusted for age, sex, and cardiovascular risk factors (BMI, hypertension, diabetes mellitus, dyslipidaemia, smoking history, regular alcohol intake, and physical activity). Figure 1A shows HRs for all-cause mortality, Figure 1B shows MACE, and 1C shows HF risk.

After a median [interquartile range (IQR)] follow-up of 2.7 (1.9–4.1) years, 413 (0.9%) participants died. A higher LV mass (indexed and non-indexed) or lower LVEF, LVCF, LVGFI, absolute GLS, and GLASED were associated with a greater incidence of death in both models (see Supplementary data online, *Table S3A*). GLASED had the largest magnitude of effect size [HR 1.38, confidence interval (CI): 1.13–1.68, *P* = 0.001] in predicting death in comparison with the next strongest marker, GLS (HR = 1.09, 95% CI: 1.04–1.15, *P* = 0.0007; see Supplementary data online, *Tables S3A*, *S4*, and *Figure 1A*, Model 1). Only modest effect sizes were detected for the other LV markers (the mean HR ranged from 1.01 to 1.09 per SD change). LV cavity size, wall stress, pressure–strain product, stroke work, stroke work indexed to the BSA, stroke work indexed to height^2.7^, GLASE, GLASE indexed to BSA, and GLASE indexed to height^2.7^ did not predict all-cause mortality.

### Prognostic associations with major adverse cardiovascular events

In our study population, 831 (1.8%) individuals experienced MACE over a median follow-up of 2.7 years. A greater LV mass and lower LVEF, LVCF, LVGFI, absolute GLS, and GLASED were consistently associated with a greater risk of incident MACE across both models (*Figure 1B* and Supplementary data online, *Table S3B*). In Model 1, GLASED had the highest HR (1.39, CI: 1.21–1.61, *P* < 0.0001), followed by GLS (HR = 1.12, 95% CI: 1.08–1.16, *P* < 0.0001).

### Prognostic associations with heart failure

A total of 245 (0.5%) participants were hospitalized with HF after a median follow-up of 2.7 years. A greater LV end-diastolic diameter and volume, greater unindexed and indexed LV mass, and lower absolute GLS and GLASED were associated with a greater risk of incident HF in a Cox model adjusted for age and sex (*Figure 1C*, Model 1). A lower LVEF, LVCF, LVGFI, and stroke work indexed to the LV mass were associated with a greater risk of HF (see Supplementary data online, *Table S3C*). For HF risk, the LV end-diastolic diameter indexed to height^2.7^ had the largest effect size (HR = 1.45, 95% CI: 1.33–1.57, *P* < 0.0001), followed by GLASED (HR = 1.41, 95% CI: 1.06–1.88, *P* = 0.019) and GLS (HR = 1.30, 95% CI: 1.21–1.40, *P* < 0.0001).

Additional adjustment for conventional cardiovascular risk factors (Model 2) rendered the prognostic association between GLASED and HF non-significant. The other associated LV markers from Model 1 had slightly attenuated effect sizes, while retaining statistical significance in Model 2 (*Figure 1C* and Supplementary data online, *Table S3C*). Supplementary data online, *Table S4* shows a comparison of HR between GLASED and the other potential prognostic markers for HF, MACE, and all-cause mortality.

### Prognostic associations in the normal LVEF subgroup

After a subgroup analysis of individuals with a normal LVEF (defined as >55%), GLASED remained a strong predictor of MACE and all-cause mortality, with a greater HR than GLS (Model 1, HR = 1.39; CI: 1.18–1.64; *P* < 0.0001 vs. HR = 1.13; CI: 1.08–1.18; *P* < 0.0001 for MACE and Model 1, HR = 1.38; CI: 1.11–1.73; *P* = 0.005 vs. HR = 1.10; CI: 1.03–1.17; *P* = 0.003 for all-cause mortality); however, the LVEF was not significantly associated with these outcomes (see Supplementary data online, *Table S5A*–*C*). Other LV markers were either not predictive or had a small effect size (mean HR range: 1.01–1.09) for MACE and all-cause mortality. For HF, the LVEF and GLASED were not associated with incident events, but the LV mass, LV end-diastolic volume, and GLS remained significantly associated with incident events.

### AIC ranking

The model fit assessment demonstrated that GLASED had the best predictive performance for all-cause mortality according to the AIC compared with the other LV markers (*Table 1*). The GLASED ranking was followed by GLASE/BSA, GLASE/height^2.7^, GLASE, the pressure–strain product, GLS, and Lamé wall stress (*Table 1*). Notably, the *C*-statistics, which indicate the model’s discriminative performance, of different LV markers were broadly comparable in the models for all-cause mortality (see Supplementary data online, *Table S3A*–*C*). There was no evidence of violation of the proportional hazard assumption in the Cox models on inspection of the Shoenfeld residuals. With MACE, GLASED had the best prediction model that fit the data best, followed by GLASE/height^2.7^, GLASED/BSA, the pressure–strain product, GLS, and the Lamé wall stress (*Table 2*). For HF, GLASE/height^2.7^, GLASE/BSA, and GLASE, followed by GLASED, GLS, pressure–strain product, and Lame longitudinal stress, performed the best (*Table 3*).

**Table 1:** Model performance rankings for all-cause mortality events in fully adjusted Cox models (Model 2) by the AIC, where lower values represent a better model fit

Rank	LV marker	AIC	ΔAIC
1	GLASED	6617.91	0.00
2	GLASE indexed to BSA	6622.05	4.14
3	GLASE indexed to height^2.7^	6622.11	4.20
4	GLASE	6622.38	4.46
5	Pressure–strain product	6733.41	115.50
6	GLS	6815.18	197.27
7	LV Lamé’s wall stress	7030.60	412.68
8	Stroke work indexed to LV mass	7187.65	569.73
9	Stroke work indexed to height^2.7^	7194.94	577.02
10	Stroke work indexed to BSA	7195.09	577.17
11	Stroke work	7195.18	577.26
12	LV global functional index	7270.04	652.12
13	LVEF	7271.80	653.88
14	LVCF	7274.57	656.66
15	LV mass indexed to BSA	7275.81	657.90
16	LV mass indexed to height^2.7^	7276.51	658.60
17	LV mass	7276.96	659.04
18	LV end-diastolic volume indexed to BSA	7280.08	662.16
19	LV end-diastolic volume indexed to height^2.7^	7280.54	662.62
20	LV end-diastolic volume	7281.07	663.15
21	LV end-diastolic diameter indexed to BSA	7281.53	663.62
22	LV end-diastolic diameter	7281.79	663.88
23	LV end-diastolic diameter indexed to height^2.7^	7284.23	666.32

**Table 2:** Model performance rankings for major adverse cardiovascular events in fully adjusted Cox models (Model 2) by the AIC, where lower values represent a better model fit

Rank	LV marker	AIC	ΔAIC
1	GLASED	13 676.91	0.00
2	GLASE indexed to height^2.7^	13 679.03	2.11
3	GLASE indexed to BSA	13 679.61	2.70
4	GLASE	13 680.00	3.08
5	Pressure–strain product	13 988.92	312.01
6	GLS	14 227.83	550.92
7	LV Lamé’s wall stress	14 683.49	1006.58
8	Stroke work indexed to LV mass	14 955.13	1278.22
9	Stroke work indexed to height^2.7^	14 964.86	1287.95
10	Stroke work indexed to BSA	14 965.22	1288.30
11	Stroke work	14 966.18	1289.27
12	LV mass indexed to BSA	15 152.64	1475.73
13	LV mass indexed to height^2.7^	15 155.30	1478.39
14	LV mass	15 172.87	1495.96
15	LVCF	15 192.90	1515.98
16	LV end-diastolic diameter	15 212.99	1536.08
17	LV end-diastolic diameter indexed to BSA	15 213.00	1536.09
18	LV global functional index	15 213.15	1536.24
19	LV end-diastolic diameter indexed to height^2.7^	15 215.50	1538.59
20	LVEF	15 236.48	1559.57
21	LV end-diastolic volume indexed to height^2.7^	15 238.68	1561.77
22	LV end-diastolic volume indexed to BSA	15 241.71	1564.80
23	LV end-diastolic volume	15 246.06	1569.14

**Table 3:** Model performance rankings for heart failure in fully adjusted Cox models (Model 2) by the AIC, where lower values represent a better model fit

Rank	LV marker	AIC	ΔAIC
1	GLASE indexed to height^2.7^	3080.57	0.00
2	GLASE indexed to BSA	3080.61	0.03
3	GLASE	3081.51	0.94
4	GLASED	3087.01	6.44
5	GLS	3222.70	142.13
6	Pressure–strain product	3223.46	142.89
7	LV Lamé’s wall stress	4078.12	997.55
8	LVEF	4177.55	1096.98
9	LV global functional index	4188.85	1108.28
10	LV end-diastolic diameter	4217.67	1137.09
11	Stroke work indexed to LV mass	4218.17	1137.60
12	LV end-diastolic volume indexed to BSA	4218.58	1138.01
13	LV end-diastolic volume	4226.51	1145.93
14	LV mass indexed to BSA	4226.83	1146.26
15	LV end-diastolic volume indexed to height^2.7^	4226.93	1146.36
16	LV mass indexed to height^2.7^	4232.60	1152.03
17	LV mass	4234.23	1153.66
18	LV end-diastolic diameter indexed to BSA	4237.61	1157.04
19	Stroke work	4263.24	1182.67
20	Stroke work indexed to BSA	4263.36	1182.79
21	Stroke work indexed to height^2.7^	4263.41	1182.84
22	LVCF	4268.40	1187.83
23	LV end-diastolic diameter indexed to height^2.7^	4282.04	1201.46

### Kaplan–Meier cumulative hazards of potential prognostic markers

**Figure 2:**
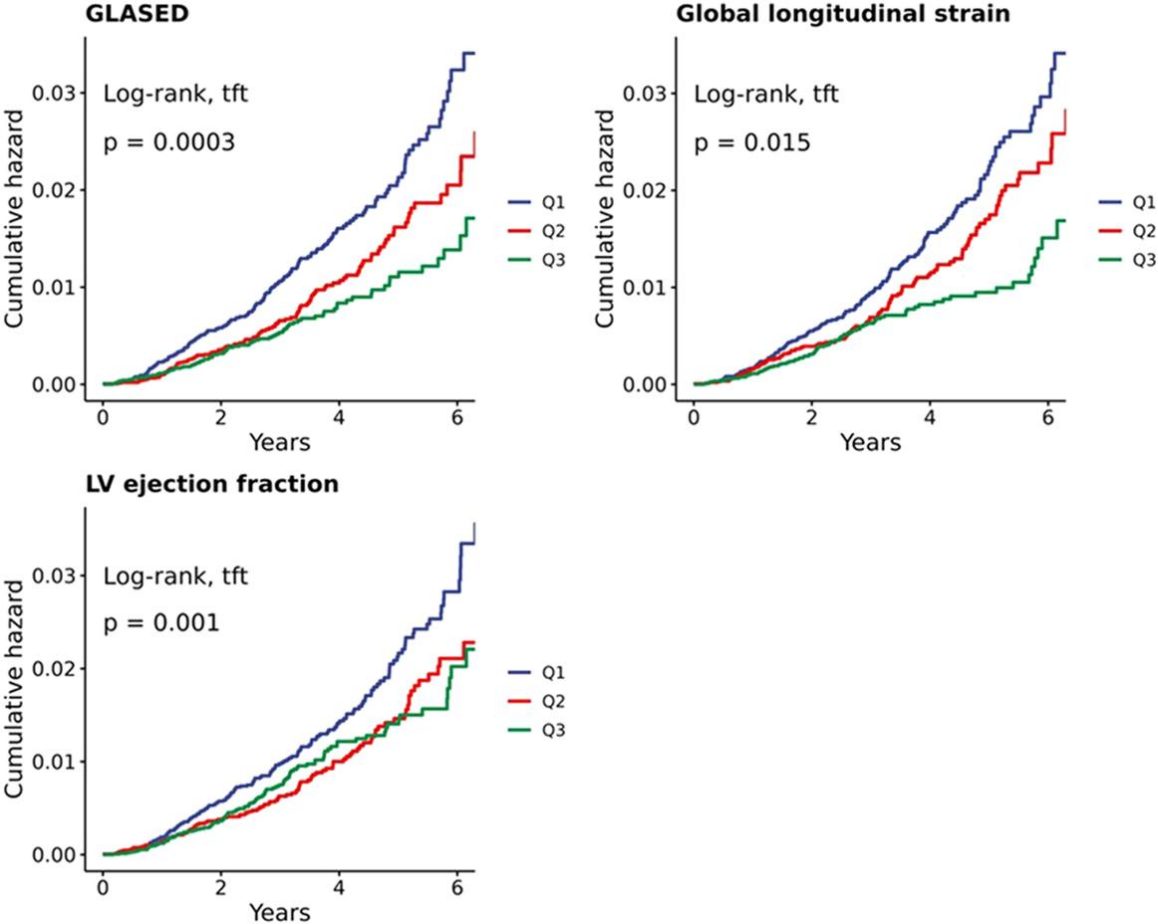
Kaplan–Meier cumulative hazard curves with potential prognostic markers in tertiles for all-cause mortality (see Supplementary data online, *Figure S4* for all results).

The unadjusted Kaplan–Meier cumulative hazards for all-cause mortality for GLASED, GLS, and LVEF are shown in *Figure 2*, and all-cause mortality, MACE, and HF risk for all the markers are shown in height^2.7^ (see Supplementary data online, *Figure S4A–C*). The lowest tertile of the LVEF had the highest number of MACE (*P* < 0.0001), HF (*P* < 0.0001), and all-cause mortality (*P* = 0.001), but there was no difference between the other two tertiles for any event.

An analysis of the GLS revealed differences in all three tertiles for MACE (*P* = 1 × 10^−4^) and all-cause mortality (*P* = 0.015) but did not reveal differences in HF risk in two of the three tertiles (*Figure 2* and Supplementary data online, *Figure S4*). The lowest stress tertile was also linked to the highest risk of MACEs (*P* < 0.00001) and all-cause mortality (*P* = 0.014) but did not predict HF risk (*P* = 0.7). The pressure–strain product did not predict the risk of either all-cause mortality or MACE; although HF risk was significant (*P* = 0.0075), it did not differ between the upper two tertiles.

Stroke work was associated with a greater risk of MACEs in the highest tertile (*P* = 0.02) but not with HF risk or all-cause mortality. Stroke work indexed to height^2.7^ or BSA did not predict future risk. Stroke work indexed to the LV mass showed that the risk of MACE (*P* < 0.0001), HF risk (*P* < 0.0001), and all-cause mortality (*P* = 0.001) were the greatest in the lowest tertile only (*P* < 0.001) and did not distinguish between Q2 and Q3 for any outcome (see Supplementary data online, *Figure S4*).

The hard endpoint of all-cause mortality (primary endpoint) was best identified by GLASED, with the lowest *P*-value (*P* = 0.0003) combined with good separation between tertiles (*Figure 2* and Supplementary data online, *Figure S4*). According to *post hoc* analysis, the GLASED was significantly lower in individuals diagnosed with atrial fibrillation (see Supplementary data online, *Table S6*).

### Correlation of the potential prognostic markers

There were significant correlations between different LV markers (see Supplementary data online, *Figure S3*). The strongest correlation was observed between the LVEF and the LVGFI (*r* = 0.93, *P* < 0.0001) and between GLASE/height^2.7^ and GLASE/BSA (*r* = 0.93, *P* < 0.0001). GLASED was strongly correlated with stroke work indexed to the LV mass (*r* = 0.8); moderately correlated with the GLS, LVCF, and LVGFI (*r* = 0.69, 0.64, and 0.5, respectively); and weakly correlated with LVEF (*r* = 0.3). The stress and strain were weakly correlated (*r* = 0.24, *P* < 0.05).

## Discussion

To our knowledge, this is the first study to perform a comprehensive comparative analysis of potentially important prognostic markers of LV structure and contractile function in a large community-based cohort consisting of individuals with a mostly normal LV structure (such as wall thickness and dimensions) and ejection fraction.

### Sex differences

In accordance with the findings of previous studies,^25,26^ we found a greater LVEF in females despite their lower wall thickness. Modelling suggested that this finding is a consequence of the lower end-diastolic diameter in females.^17,27^ LV wall stress, the absolute GLS, and the GLASED were greater in females. This result contrasts with our echocardiographic study in which young male athletes had a greater GLASED than female athletes.^17^ These sex differences were no longer present in the veteran athletes (mean age was 54 years). We speculate that the lower GLASED in males in this study reflects their greater age (mean 64 years) and corresponding greater incidence of CVD.

### Predictor markers for all-cause mortality, MACEs, and heart failure

An increase in the LV mass slightly increased the risk of all outcomes, although an increase in the LV mass indexed to height^2.7^ improved the prediction. The LVEF predicted HF risk to a modest extent but was a weak marker for assessing the risk of MACE or all-cause mortality. The unreliable nature of the LVEF stems from the effects of underlying variables that modulate it, such as changes in myocardial structure and strain.^16,27–30^ A greater wall thickness or lower end-diastolic diameter increases the LVEF independently of myocardial strain.^12,16,27–30^ The GLS performed better than the LVEF in predicting the risk of HF, MACE, and all-cause mortality. GLASED was the strongest marker for predicting the risk of MACEs and all-cause mortality (HR ≈ 1.4). The larger HR of GLASED compared with that of the GLS may reflect the impact of stress on the latter because GLASED corrects the GLS for afterload. The magnitude of the effect of GLASED for HF prediction was comparable with that of the GLS (HR 1.41 vs. 1.30). This finding may be a consequence of the inclination of clinicians to be more likely to diagnose HF in the presence of a dilated ventricle or reduced myocardial strain. GLASED is obtained by multiplying GLS by LV stress. In dilated ventricles, stress will increase, and thus, the GLASED will increase relative to the GLS. If dilated ventricles are overrepresented in the HF group, GLASED may be slightly overestimated, which would reduce its prediction power of HF.

There are theoretical reasons supporting the superiority of GLASED over other potential risk markers. GLASED inherently adjusts for both geometric changes and ventricular pressure. We posit that myocardial stress is a more accurate measure of afterload compared with BP alone. Cardiomyocytes primarily respond to LV stress rather than LV pressure, suggesting that the pressure–strain product might be less reliable than GLASED. The pressure–strain loop itself cannot directly quantify myocardial work; geometric data are essential for this purpose. Work is defined as force multiplied by distance, and work density is derived from the stress–strain loop, not the pressure–strain loop. Substituting luminal pressure for myocardial stress, as in the pressure–strain loop calculation, is inaccurate because: (i) luminal pressure exerts a force perpendicular to the stress experienced by the myocardium, and (ii) it is stress, not luminal pressure, that is sensed by the cardiomyocytes. Pressure–strain loops serve as an indirect index of work and are only directly comparable when ventricular geometry remains constant. Consequently, the pressure–strain product/loop is expected to be less informative in cohorts exhibiting significant eccentric or concentric remodelling. In support of this, we have previously demonstrated that pressure–strain product was a less reliable predictor of mortality compared with GLASED in patient groups with substantial remodelling.^1^ Similarly, the limited prognostic value of LVCF, LVGFI, and stroke work might be attributed to their inability to account for the influence of geometric changes due to dilation and wall thickness on stress.

A lower GLASED was associated with established cardiovascular risk factors, such as smoking, diabetes, elevated body mass index (BMI), hypertension, and hyperlipidaemia. This association arises because each of these factors independently affects both myocardial stress and strain. Since Model 2 incorporated these risk factors individually, the observed correlation between these factors and contractile stress/strain might explain the reduced predictive power of Model 2. This highlights the potential strength of the GLASED as a comprehensive marker that integrates the influence of conventional cardiovascular risk factors on clinical outcomes.

The LV diastolic diameter indexed to height^2.7^ was strongly predictive of HF, which may indicate the presence of HF with a cause not primarily due to LV myocardial dysfunction, such as that due to left-sided valve regurgitation. Heart failure also occurs in patients with non-LV myocardial disorders, such as right HF, where the GLASED is expected to be normal. Furthermore, this finding should be treated with caution because the LV diastolic diameter indexed to height^2.7^ was ranked last on the AIC (23rd) and was not significant according to the Kaplan–Meier curve (*P* = 0.62). Furthermore, none of the other measures, such as the LV diastolic diameter indexed to BSA or LV volume markers, performed well. The diagnosis of HF can be subjective, and factors such as myocardial strain and internal chamber dimensions obtained through imaging techniques can further influence diagnostic decisions. Heart failure risk may, therefore, represent a softer endpoint than MACE and all-cause mortality.

### Limitations and future directions

Our cohort had a low pre-test probability of events given their low rates of comorbidities and structural abnormalities. For example, in the UK Biobank cohort, only 2% of individuals had an LV wall thickness >13 mm, which could have resulted in an underpowered assessment in this group. We found GLASED to be even more useful in predicting the expected prognosis in the presence of greater structural abnormalities, such as amyloid heart disease and dilated cardiomyopathy.^1^ Specifically designed prospective studies will be needed to assess the role of the GLASED and GLASE in predicting the risk of all adverse cardiovascular events.

GLASED is an approximation of myocardial longitudinal contractance derived using the area under the stress–strain curve^14^ but requires more sophisticated analyses that are currently unsuitable for clinical practice.^1^ However, GLASED gives comparable results with those of an area under the curve method.^1,17^ Future work should involve the automated calculation of GLASED, perhaps using machine learning algorithms with echocardiography, to allow widespread application.

The calculations of stress and the derived measurements of GLASED are prone to propagation error, emphasizing that accurate measurements of wall thickness and end-diastolic diameter are important.

Ambulatory BP data may improve the accuracy but were not available for this cohort. One advantage of our study is that it showed positive results with brachial BP cuff measurements, so it is more easily applicable in clinical practice. No ‘diastolic function’ tests were performed, as this study principally aimed to assess geometry and systolic function, and the accuracy of diastolic function assessment using CMR is questionable. Strain rates were not assessed because frame rates were too low for accurate results. Finally, information on major adverse cardiovascular events and HF was obtained from hospital admission records with potential encoding errors. For HF, we were unable to correlate our findings with more sensitive biomarkers, such as BNP, due to data unavailability.

While acknowledging the limitations of CMR for routine risk assessment in community settings, we propose that future investigations explore the potential of GLASED as a prognostic tool in broader patient populations. This could include cohorts with post-myocardial infarction, valvular disease, and hypertrophic cardiomyopathy, potentially using a more readily available modality like echocardiography.

### Clinical perspective

Identifying the most powerful LV imaging marker(s) for assessing the outlook is crucial for understanding the pathophysiological mechanism of heart failure and exercise intolerance, guiding consensus documents, best patient management, and designing clinical intervention trials. GLASED overcomes the limitations of both GLS and LVEF by incorporating afterload correction and provides better prognostic information than current markers. The GLASED is applicable to both CMR and echocardiography^17^ and is easily and rapidly calculated from only four input variables.

GLASED offers a more direct assessment of myocardial function than prior methods. This advantage stems from its use of four key features: (i) direct myocardial data acquisition (rather than relying on luminal measurements), (ii) estimation of energy production per unit of myocardial tissue, (iii) incorporation LV geometry and systolic pressure, and (iv) integration of afterload correction of strain. Given these capabilities, GLASED has the potential to be valuable in evaluating the severity of LV myocardial dysfunction in various cardiac conditions, including valvular heart disease. A better risk stratification can be obtained by incorporating GLASED into cardiac imaging studies.

## Conclusion

This exploratory analysis assessed the potential role of 23 different structural and functional prognostic markers of the LV and compared them with a new measure of contractile function called GLASED. GLASED is simple and quick to calculate from the mean wall thickness, end-diastolic diameter, systolic BP, and GLS. We showed that the LVEF and multiple other potential LV risk markers are of limited value in predicting patient prognosis. Despite our cohort consisting of low-risk individuals, GLASED improved the risk assessment of both major adverse cardiovascular events and all-cause mortality compared with other imaging measurements.

## Supplementary data

Supplementary data are available at *European Heart Journal – Cardiovascular Imaging* online.

## Author contributions

S.C. derived the strain measurements. N.A. performed the extraction of all other image-derived markers and statistical analyses. D.H.M. conceived the project and wrote the first draft. H.Z. provided essential engineering and physics expertise. S.E.P. had a supervisory role, helped with the study design, and performed major manuscript edits. All the authors contributed to the manuscript, provided critical feedback, agreed to the manuscript, and have equal responsibility for its content.

## Supplementary Material

jeae133_Supplementary_Data

## Data Availability

The UK Biobank will make the data available to all bona fide researchers for all types of health-related research that is in the public interest, without preferential or exclusive access for any persons. All researchers will be subject to the same application process and approval criteria as specified by the UK Biobank. For more details on the access procedure, see the UK Biobank website: http://www.ukbiobank.ac.uk/register-apply/. Other information, including a prewritten Excel template for calculating GLASE and GLASED, is available from the corresponding author upon reasonable request.

## NHS Digital

Researchers using data from NHS Digital must cite the copyright of NHS Digital as follows: ‘Copyright^©^ (year), NHS Digital. Re-used with the permission of the NHS Digital [and/or UK Biobank]. All rights reserved.’ Where practicable, outputs cite the source of the data as ‘this work uses data provided by patients and collected by the NHS as part of their care and support.’

## Public Health Scotland

Attribution statement—Please acknowledge National Safe Haven for providing access to the data assets and the environment, which were made available as part of the Data and Connectivity National Core Study Programme in papers and other research outputs. For example, this research used data assets made available by National Safe Haven as part of the Data and Connectivity National Core Study, led by Health Data Research UK in partnership with the Office for National Statistics and funded by UK Research and Innovation (research that commenced between 1 October 2020 and 31 March 2021 grant Ref. MC_PC_20029; 1 April 2021 and 30 September 2022 grant Ref. MC_PC_20058).
