# Supplementary material for: Global longitudinal active strain energy density (GLASED): a powerful prognostic marker in a community-based cohort
Source: Eur Heart J Cardiovasc Imaging. 2024 May 20;25(10):1405–14. doi: 10.1093/ehjci/jeae133 (PMC11441035; doi:10.1093/ehjci/jeae133)
Supplement: jeae133_Supplementary_Data [file jeae133_supplementary_data.pdf]

gSupplementary data

Index

Statistical analysis

Figures

Figure S1. Distribution of potential prognostic markers

Figure S2. Relationships between potential prognostic markers and age, sex and risk factors (by univariate regression)

Figure S3A. Correlation matrix of potential prognostic markers (*r* values shown)

Figure S3B. *P* values for correlations of potential prognostic markers

Figure S4A. Kaplan-Meier cumulative hazards analysis with potential prognostic markers in tertiles for all-cause mortality

Figure S4B. Kaplan-Meier cumulative hazards analysis with potential prognostic markers in tertiles for major adverse cardiovascular events

Figure S4C. Kaplan-Meier cumulative hazards analysis with potential prognostic markers in tertiles for heart failure risk

Tables

Table S1. The ICD10 codes used to define MACEs and heart failure

Table S2A. Demographics and main results (n=44,957)

Table S2B. Baseline characteristics stratified by cardiovascular disease status

Table S3A. Cox regression analysis of potential prognostic markers for all-cause mortality (Holm–Bonferroni corrected p<0.05 in bold)

Table S3B. Cox regression analysis of potential prognostic markers for major adverse cardiovascular events (Holm–Bonferroni corrected p<0.05 in bold)

Table S3C. Cox regression analysis of potential prognostic markers for heart failure (Holm–Bonferroni corrected p<0.05 in bold)

Table S4. Comparison of hazard ratios according to GLASED vs other potential prognostic markers

Table S5A. Cox regression analysis of potential prognostic markers for all-cause mortality in the subgroup with a normal LVEF (>55%)

Table S5B. Cox regression analysis of potential prognostic markers for major adverse cardiovascular events in the subgroup with a normal LVEF (>55%)

Table S5C. Cox regression analysis of potential prognostic markers for heart failure in the subgroup with a normal LVEF (>55%)

Table S6. Atrial fibrillation and GLASED

Statistical analysis

Statistical analyses were performed using R version 4.1.1. Baseline characteristics are presented for the whole cohort and were stratified by the presence or absence of CVD. Cardiovascular disease status was ascertained from self-reported medical history taken at the time of the visit to the imaging centre and included angina, heart attack/myocardial infarction, heart failure/pulmonary oedema, arrhythmias, stroke, peripheral vascular disease, valvular heart disease, cardiomyopathy and pericardial disease and prevalent MACE and heart failure identified from hospital episode statistics (Table S1). Correlations between the potential prognostic markers were determined by calculating the Pearson correlation coefficient (r). Univariate linear regression analysis was performed to evaluate the associations between LV markers and age, sex, and conventional cardiovascular risk factors. For GLS, we used absolute (positive) values for ease of interpretation. Unadjusted associations between LV measurements stratified into tertiles and adverse outcomes (heart failure incidence, MACE incidence and all-cause mortality) were examined via cumulative hazard curves with the log-rank trend test to evaluate survival differences. We constructed Cox proportional hazards models to examine the associations between LV markers and adverse outcomes after accounting for potential confounders. In the primary analysis, the Cox model was adjusted for age and sex (Model 1). A Holm–Bonferroni-corrected *P* value of less than 0.05 was considered to indicate statistical significance in primary analyses. In the secondary sensitivity analyses, (i) we adjusted for age and sex. cardiovascular risk factors (body mass index (BMI), smoking status, regular alcohol intake, self-reported physical activity in total minutes per week, hypertension, diabetes mellitus and hyperlipidaemia) (Model 2). (ii) We repeated Model 1 and Model 2 in a subset of individuals with a normal LVEF (>55%). Cardiovascular risk factors were ascertained from self-reported medical history, secondary care records (HES data), the use of antihypertensive medications, the use of lipid lowering medication or insulin, a total cholesterol concentration ≥ 7.0 mmol/L (to define hyperlipidaemia) and a glycated haemoglobin level (HBA1C) ≥ 48 mmol/mol (to define diabetes mellitus). The level of physical activity was estimated with the International Physical Activity Questionnaire.26 Individuals who reported alcohol consumption three or more days per week were classified as regular alcohol users. We centred and scaled the variables; therefore, the effect sizes from the regression models represent the per standard deviation (SD) change in the exposure variable. The proportional hazard assumption was assessed by checking the correlation of Schoenfeld residuals with time. The differences in the hazard ratios (HRs) of the LV markers were evaluated by an unpaired t test using the bootstrap resampling technique. The performance of different LV markers in Cox models was compared by the Akaike information criterion (AIC), where smaller values indicate a better model fit and greater predictive accuracy. The discriminative

performance of the LV markers was evaluated with Uno C-statistics, where larger values suggest better performance.

Figure S1. Distribution of potential prognostic markers

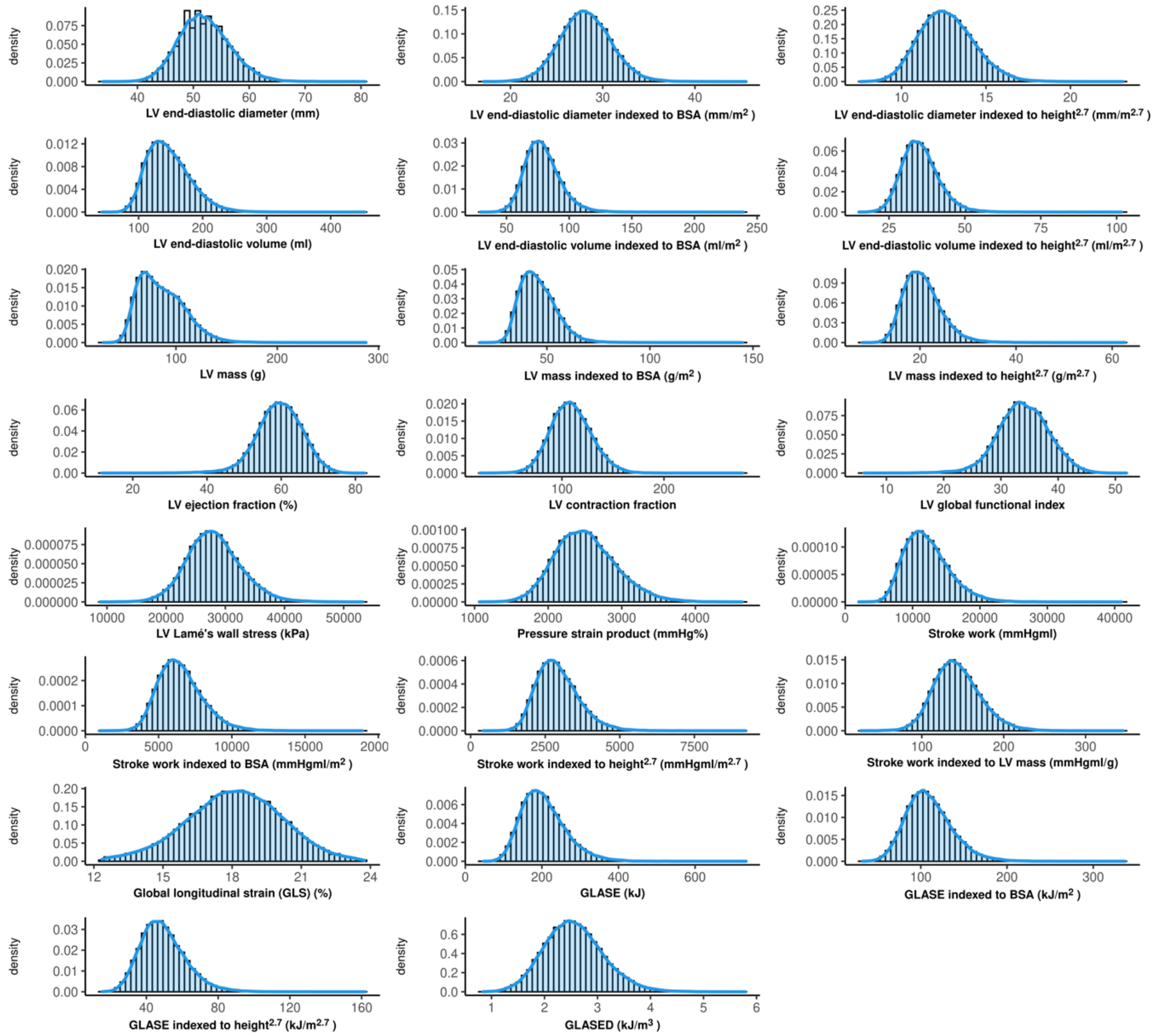

Figure S2. Relationships between potential prognostic markers and age, sex and risk factors (by univariate regression)

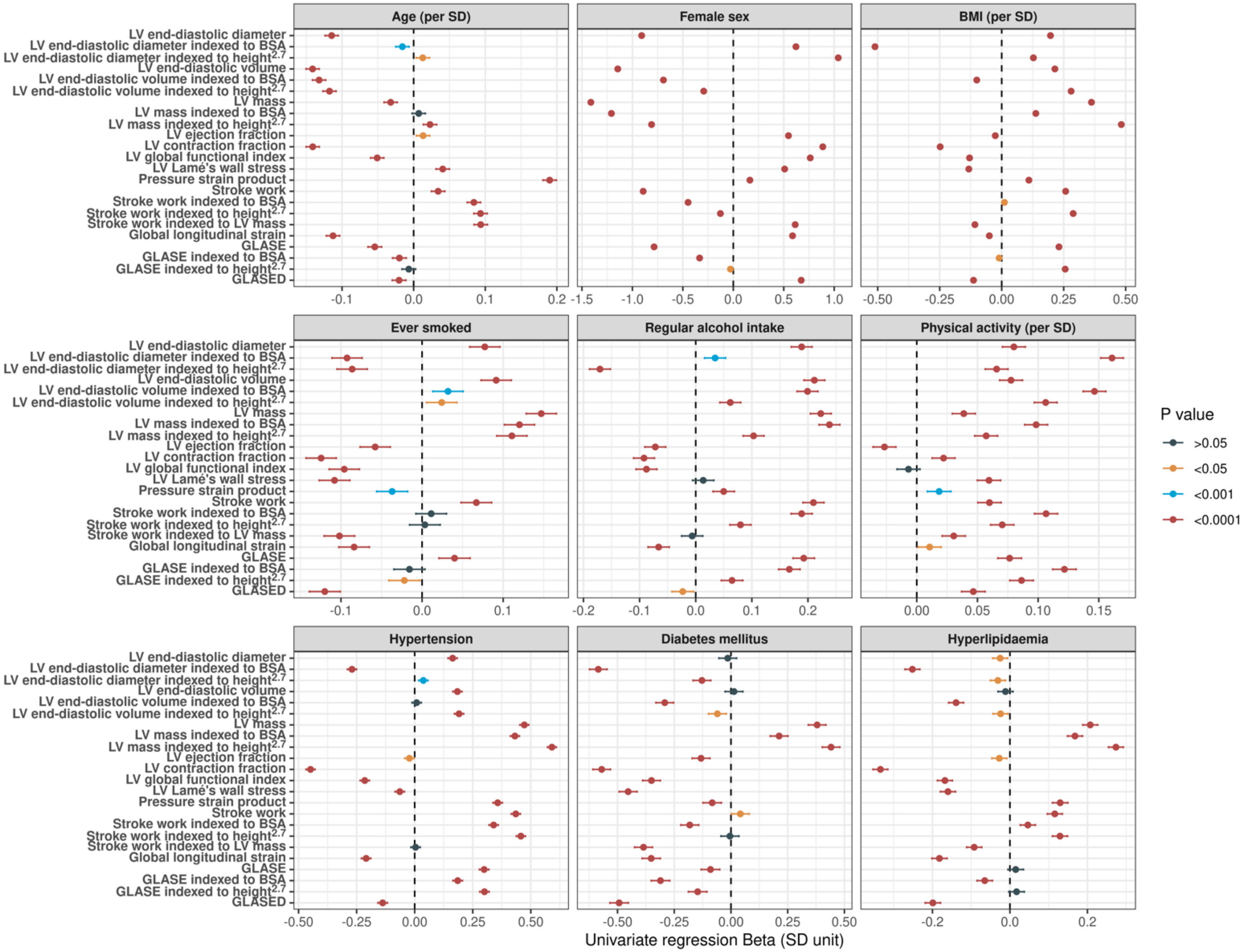

Figure S3A. Correlation matrix of potential prognostic markers (*r* values shown)

	LV mass	LV mass indexed to BSA	LV mass indexed to height^2.7	LV end−diastolic volume	LV end−diastolic diameter	LV end−diastolic volume indexed to BSA	LV end−diastolic volume indexed to height^2.7	Stroke work	GLASE	Stroke work indexed to BSA	GLASE indexed to BSA	Stroke work indexed to height^2.7	GLASE indexed to height^2.7	LV end−diastolic diameter indexed to BSA	Pressure strain product	LV Lamé's wall stress	LV end−diastolic diameter indexed to height^2.7	GLASED	Stroke work indexed to LV mass	Global longitudinal strain	LV contraction fraction	LV global functional index	LV ejection fraction
LV mass	1	0.92	0.82	0.81	0.67	0.57	0.49	0.7	0.65	0.45	0.4	0.37	0.32	−0.25	0.02	−0.22	−0.32	−0.31	−0.32	−0.29	−0.54	−0.43	−0.29
LV mass indexed to BSA	0.92	1	0.88	0.74	0.61	0.67	0.58	0.65	0.62	0.55	0.5	0.45	0.4	0.03	0.05	−0.17	−0.12	−0.27	−0.28	−0.27	−0.51	−0.41	−0.28
LV mass indexed to height^2.7	0.82	0.88	1	0.6	0.5	0.51	0.67	0.58	0.55	0.49	0.44	0.57	0.52	−0.01	0.13	−0.16	0.18	−0.23	−0.23	−0.22	−0.5	−0.36	−0.2
LV end−diastolic volume	0.81	0.74	0.6	1	0.89	0.88	0.76	0.76	0.77	0.58	0.58	0.45	0.46	0.08	0.01	0.12	−0.13	0.03	−0.01	−0.11	−0.09	−0.24	−0.3
LV end−diastolic diameter	0.67	0.61	0.5	0.89	1	0.82	0.73	0.66	0.74	0.51	0.6	0.42	0.51	0.29	0.03	0.27	0.08	0.17	0.04	−0.05	−0.02	−0.21	−0.31
LV end−diastolic volume indexed to BSA	0.57	0.67	0.51	0.88	0.82	1	0.86	0.65	0.69	0.66	0.69	0.52	0.55	0.5	0.03	0.29	0.17	0.2	0.14	−0.01	0.12	−0.11	−0.25
LV end−diastolic volume indexed to height^2.7	0.49	0.58	0.67	0.76	0.73	0.86	1	0.6	0.64	0.62	0.66	0.68	0.71	0.46	0.12	0.32	0.51	0.25	0.19	0.05	0.13	−0.06	−0.18
Stroke work	0.7	0.65	0.58	0.76	0.66	0.65	0.6	1	0.88	0.91	0.76	0.83	0.69	0	0.5	0.36	−0.08	0.28	0.43	0.04	0.05	0.13	0.16
GLASE	0.65	0.62	0.55	0.77	0.74	0.69	0.64	0.88	1	0.8	0.92	0.72	0.84	0.11	0.58	0.47	0	0.49	0.35	0.29	0.02	0	−0.02
Stroke work indexed to BSA	0.45	0.55	0.49	0.58	0.51	0.66	0.62	0.91	0.8	1	0.84	0.92	0.78	0.31	0.61	0.53	0.17	0.46	0.64	0.14	0.23	0.3	0.28
GLASE indexed to BSA	0.4	0.5	0.44	0.58	0.6	0.69	0.66	0.76	0.92	0.84	1	0.78	0.93	0.42	0.68	0.63	0.26	0.68	0.51	0.42	0.19	0.14	0.07
Stroke work indexed to height^2.7	0.37	0.45	0.57	0.45	0.42	0.52	0.68	0.83	0.72	0.92	0.78	1	0.85	0.26	0.65	0.52	0.4	0.48	0.65	0.18	0.23	0.32	0.33
GLASE indexed to height^2.7	0.32	0.4	0.52	0.46	0.51	0.55	0.71	0.69	0.84	0.78	0.93	0.85	1	0.37	0.73	0.63	0.48	0.7	0.53	0.45	0.18	0.17	0.12
LV end−diastolic diameter indexed to BSA	−0.25	0.03	−0.01	0.08	0.29	0.5	0.46	0	0.11	0.31	0.42	0.26	0.37	1	0.06	0.49	0.7	0.45	0.33	0.19	0.42	0.16	−0.04
Pressure strain product	0.02	0.05	0.13	0.01	0.03	0.03	0.12	0.5	0.58	0.61	0.68	0.65	0.73	0.06	1	0.56	0.18	0.72	0.67	0.59	0.17	0.33	0.38
LV Lamé's wall stress	−0.22	−0.17	−0.16	0.12	0.27	0.29	0.32	0.36	0.47	0.53	0.63	0.52	0.63	0.49	0.56	1	0.4	0.86	0.78	0.24	0.48	0.26	0.05
LV end−diastolic diameter indexed to height^2.7	−0.32	−0.12	0.18	−0.13	0.08	0.17	0.51	−0.08	0	0.17	0.26	0.4	0.48	0.7	0.18	0.4	1	0.41	0.32	0.23	0.33	0.2	0.07
GLASED	−0.31	−0.27	−0.23	0.03	0.17	0.2	0.25	0.28	0.49	0.46	0.68	0.48	0.7	0.45	0.72	0.86	0.41	1	0.8	0.69	0.64	0.5	0.3
Stroke work indexed to LV mass	−0.32	−0.28	−0.23	−0.01	0.04	0.14	0.19	0.43	0.35	0.64	0.51	0.65	0.53	0.33	0.67	0.78	0.32	0.8	1	0.42	0.75	0.72	0.58
Global longitudinal strain	−0.29	−0.27	−0.22	−0.11	−0.05	−0.01	0.05	0.04	0.29	0.14	0.42	0.18	0.45	0.19	0.59	0.24	0.23	0.69	0.42	1	0.55	0.59	0.5
LV contraction fraction	−0.54	−0.51	−0.5	−0.09	−0.02	0.12	0.13	0.05	0.02	0.23	0.19	0.23	0.18	0.42	0.17	0.48	0.33	0.64	0.75	0.55	1	0.85	0.6
LV global functional index	−0.43	−0.41	−0.36	−0.24	−0.21	−0.11	−0.06	0.13	0	0.3	0.14	0.32	0.17	0.16	0.33	0.26	0.2	0.5	0.72	0.59	0.85	1	0.93
LV ejection fraction	−0.29	−0.28	−0.2	−0.3	−0.31	−0.25	−0.18	0.16	−0.02	0.28	0.07	0.33	0.12	−0.04	0.38	0.05	0.07	0.3	0.58	0.5	0.6	0.93	1

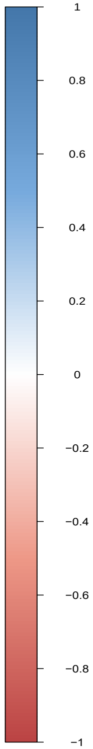

Figure S3B. *P* values for correlations of potential prognostic markers

Potential prognostic marker	LVEDd	LVEDd/BSA	LVEDd/H2.7	LV EDV	LVEDV/BSA	LV EDV/H2.7	LV mass	LV mass/BSA	LV mass/H2.7	LV ejection fraction	LV contr. fraction	LV GFI	LV Lamé's wall stress	Pressure-strain product	Stroke work	Stroke work/BSA	Stroke work/H2.7	Stroke work/LV mass	GLS	GLASE	GLASE/BSA	GLASE/H2.7	GLASED
LVEDd		7.05E-01	9.88E-02	2.93E-15	2.39E-14	2.62E-09	7.20E-08	5.08E-08	2.55E-06	4.29E-08	9.03E-05	1.84E-07	3.18E-01	8.27E-02	2.31E-05	4.60E-03	6.70E-02	1.50E-03	7.46E-05	2.03E-06	1.62E-03	3.45E-02	1.55E-02
LVEDd/BSA			4.42E-07	2.25E-01	7.67E-01	4.92E-01	1.88E-02	8.18E-02	9.95E-02	7.82E-01	2.90E-02	4.44E-01	3.25E-03	8.42E-01	3.66E-02	4.85E-01	7.07E-01	1.22E-01	3.29E-01	1.86E-01	7.64E-01	5.16E-01	2.05E-02
LVEDd/ $H^{2.7}$				1.49E-02	2.81E-01	9.79E-01	2.04E-03	1.51E-02	8.01E-02	4.83E-01	1.95E-02	1.48E-01	8.54E-03	5.33E-01	5.84E-03	2.39E-01	9.78E-01	5.06E-02	9.88E-02	3.72E-02	7.28E-01	4.51E-01	1.15E-02
LV EDV					1.57E-11	1.05E-07	2.56E-11	1.14E-10	8.93E-08	1.67E-06	9.89E-06	3.04E-07	1.10E-01	8.68E-02	7.40E-07	2.10E-03	4.80E-02	5.62E-04	2.12E-05	6.60E-07	3.54E-03	6.35E-02	2.74E-03
LVEDV/BSA						8.36E-12	2.97E-06	4.76E-07	1.08E-05	2.41E-07	7.80E-04	2.98E-06	4.51E-01	6.34E-02	1.04E-04	2.97E-03	4.77E-02	4.72E-03	1.04E-04	2.27E-05	1.52E-03	3.06E-02	2.70E-02
LV EDV/$H^{2.7}$							3.96E-05	3.96E-06	2.55E-06	7.00E-07	1.04E-03	6.49E-06	6.01E-01	1.23E-01	5.87E-04	3.95E-03	1.21E-02	9.25E-03	3.04E-04	1.22E-04	1.40E-03	5.61E-03	5.22E-02
LV mass								4.87E-18	3.51E-12	2.76E-05	4.19E-10	5.15E-08	7.93E-03	1.22E-01	8.36E-07	5.52E-03	5.63E-02	2.04E-05	4.55E-06	6.75E-06	2.11E-02	1.38E-01	5.76E-05
LV mass/BSA									2.12E-15	5.05E-06	9.55E-11	3.84E-09	1.13E-02	9.92E-02	4.27E-06	5.07E-03	4.35E-02	1.23E-05	1.25E-06	1.68E-05	1.62E-02	9.96E-02	5.98E-05
LV mass/$H^{2.7}$										1.70E-05	4.01E-11	1.55E-08	1.21E-02	1.55E-01	2.67E-05	7.42E-03	2.07E-02	2.19E-05	4.11E-06	8.02E-05	2.15E-02	5.66E-02	9.88E-05
LV ejection fraction											6.94E-06	2.96E-13	2.01E-01	1.49E-02	2.39E-02	2.35E-01	5.58E-01	2.38E-05	1.78E-05	3.27E-03	4.72E-02	1.74E-01	5.96E-03
LV contr. fraction												1.64E-10	1.66E-03	9.46E-02	1.40E-03	1.13E-01	2.58E-01	1.11E-07	4.30E-06	1.60E-03	1.35E-01	3.08E-01	7.56E-06
LV GFI													3.14E-02	2.36E-02	5.05E-03	1.56E-01	3.97E-01	2.48E-07	9.44E-07	1.27E-03	6.43E-02	2.10E-01	2.29E-04
LV Lamé's wall stress														5.97E-04	6.49E-01	2.41E-01	1.16E-01	1.30E-06	8.92E-03	8.77E-01	4.55E-02	1.38E-02	1.36E-09
Pressure-strain product															5.77E-01	6.65E-02	1.14E-02	8.63E-05	7.52E-04	3.62E-01	2.48E-02	3.08E-03	3.20E-05
Stroke work																3.69E-09	1.63E-05	1.93E-01	8.32E-03	8.13E-11	2.75E-05	1.57E-03	1.34E-01
Stroke work/BSA																	6.97E-11	7.19E-01	1.55E-01	3.64E-07	1.66E-07	9.71E-06	9.22E-01
Stroke work/$H^{2.7}$																		3.61E-01	4.11E-01	1.12E-04	4.43E-06	2.96E-07	5.31E-01
Stroke work/LV mass																			2.28E-05	1.88E-01	7.91E-01	4.19E-01	1.71E-09
GLS																				5.17E-02	5.93E-01	9.48E-01	8.48E-07
GLASE																					8.61E-09	2.00E-05	4.28E-01
GLASE/BSA																						1.94E-11	3.12E-01
GLASE/$H^{2.7}$																							1.11E-01
GLASED																							
	P values	0	0.025	0.05	0.01	0.0001																	

Figure S4A. Kaplan-Meier cumulative hazards analysis with potential prognostic markers in tertiles for all-cause mortality

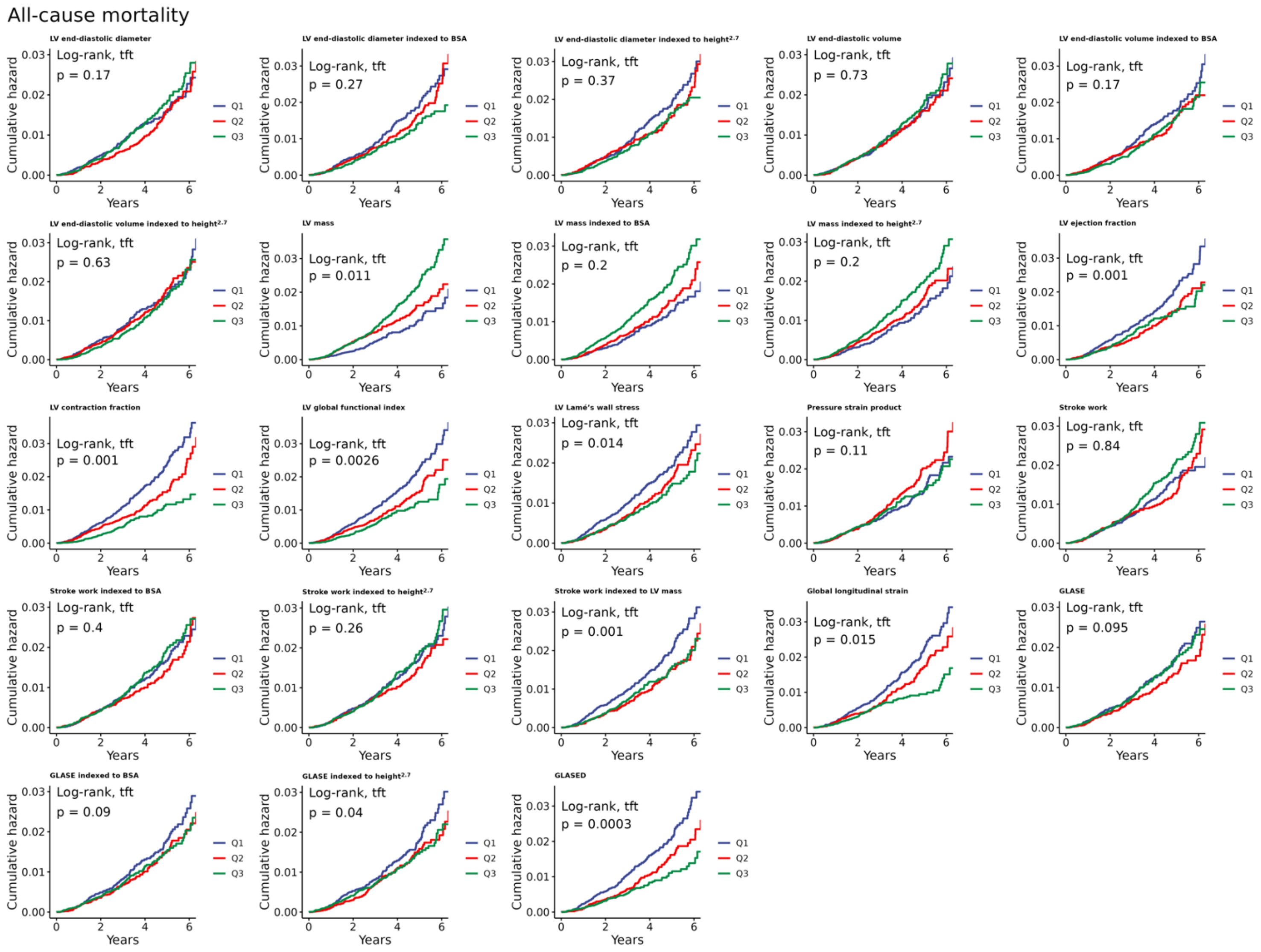

Figure S4B. Kaplan-Meier cumulative hazards analysis with potential prognostic markers in tertiles for major adverse cardiovascular events

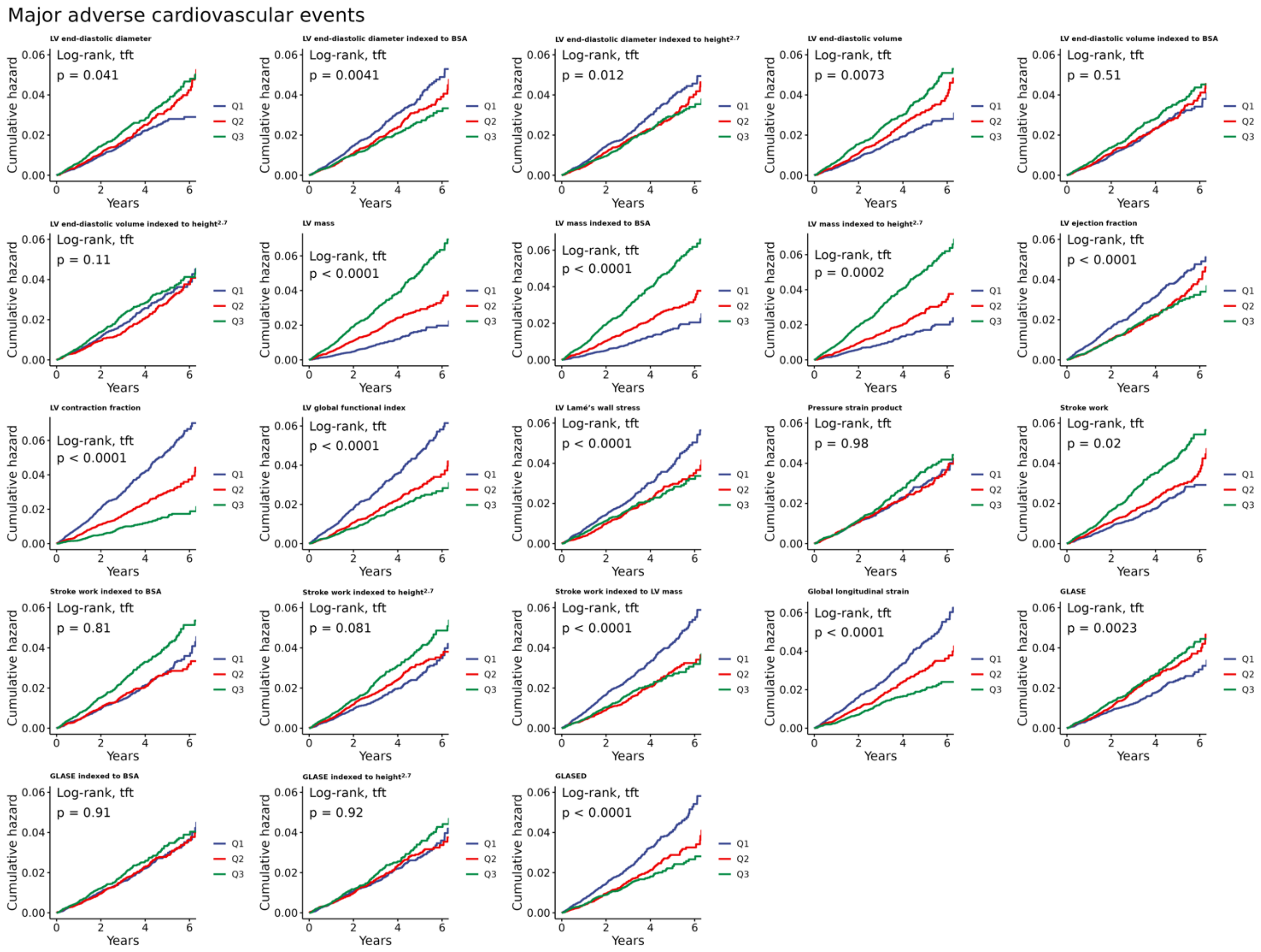

Figure S4C. Kaplan-Meier cumulative hazards analysis with potential prognostic markers in tertiles for heart failure risk

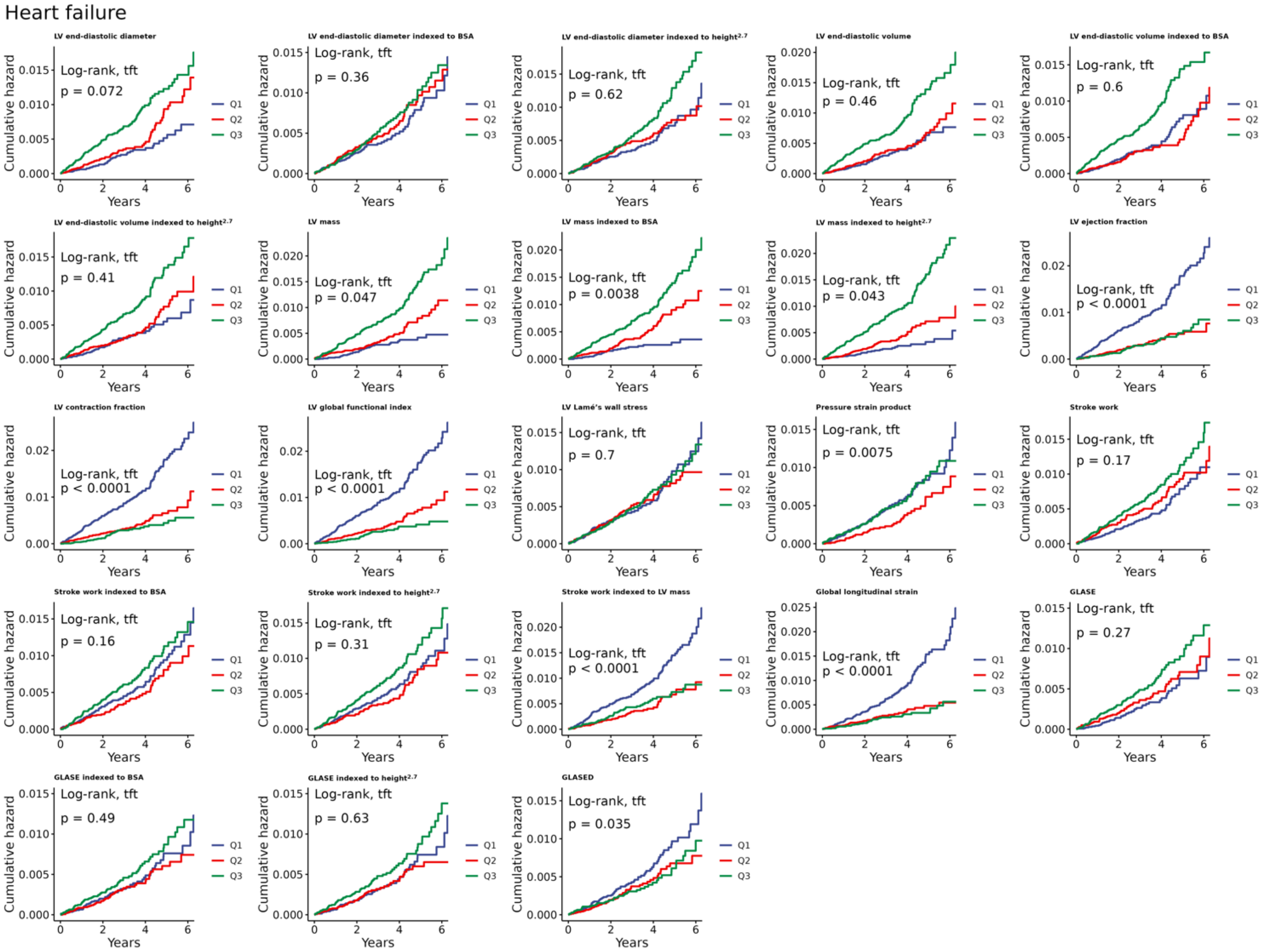

Table S1. The ICD10 codes used to define MACEs and heart failure

Condition	Coding	Meaning
**MACE**	I21	I21 Acute myocardial infarction
**MACE**	I210	I21.0 Acute transmural myocardial infarction of anterior wall
**MACE**	I211	I21.1 Acute transmural myocardial infarction of inferior wall
**MACE**	I212	I21.2 Acute transmural myocardial infarction of other sites
**MACE**	I213	I21.3 Acute transmural myocardial infarction of unspecified site
**MACE**	I214	I21.4 Acute subendocardial myocardial infarction
**MACE**	I219	I21.9 Acute myocardial infarction, unspecified
**MACE**	I21X	I21.X Presumed acute myocardial infarction (unconfirmed)
**MACE**	I22	I22 Subsequent myocardial infarction
**MACE**	I220	I22.0 Subsequent myocardial infarction of anterior wall
**MACE**	I221	I22.1 Subsequent myocardial infarction of inferior wall
**MACE**	I228	I22.8 Subsequent myocardial infarction of other sites
**MACE**	I229	I22.9 Subsequent myocardial infarction of unspecified site
**MACE**	I23	I23 Certain current complications following acute myocardial infarction
**MACE**	I230	I23.0 Haemopericardium as current complication following acute myocardial infarction
**MACE**	I231	I23.1 Atrial septal defect as current complication following acute myocardial infarction
**MACE**	I232	I23.2 Ventricular septal defect as current complication following acute myocardial infarction
**MACE**	I233	I23.3 Rupture of cardiac wall without haemopericardium as current complication following acute myocardial infarction
**MACE**	I234	I23.4 Rupture of chordae tendineae as current complication following acute myocardial infarction
**MACE**	I235	I23.5 Rupture of papillary muscle as current complication following acute myocardial infarction
**MACE**	I236	I23.6 Thrombosis of atrium, auricular appendage and ventricle as current complications following acute myocardial infarction
**MACE**	I238	I23.8 Other current complications following acute myocardial infarction
**MACE**	I24	I24 Other acute ischaemic heart diseases
**MACE**	I240	I24.0 Coronary thrombosis not resulting in myocardial infarction
**MACE**	I241	I24.1 Dressler's syndrome
**MACE**	I248	I24.8 Other forms of acute ischaemic heart disease
**MACE**	I249	I24.9 Acute ischaemic heart disease, unspecified
**MACE**	I25	I25 Chronic ischaemic heart disease
**MACE**	I250	I25.0 Atherosclerotic cardiovascular disease, so described
**MACE**	I251	I25.1 Atherosclerotic heart disease
**MACE**	I252	I25.2 Old myocardial infarction
**MACE**	I253	I25.3 Aneurysm of heart
**MACE**	I254	I25.4 Coronary artery aneurysm
**MACE**	I255	I25.5 Ischaemic cardiomyopathy
**MACE**	I256	I25.6 Silent myocardial ischaemia
**MACE**	I258	I25.8 Other forms of chronic ischaemic heart disease
**MACE**	I630	I63.0 Cerebral infarction due to thrombosis of precerebral arteries
**MACE**	I631	I63.1 Cerebral infarction due to embolism of precerebral arteries
**MACE**	I632	I63.2 Cerebral infarction due to unspecified occlusion or stenosis of precerebral arteries
**MACE**	I633	I63.3 Cerebral infarction due to thrombosis of cerebral arteries
**MACE**	I634	I63.4 Cerebral infarction due to embolism of cerebral arteries

**MACE**	I635	I63.5 Cerebral infarction due to unspecified occlusion or stenosis of cerebral arteries
**MACE**	I636	I63.6 Cerebral infarction due to cerebral venous thrombosis, nonpyogenic
**MACE**	I638	I63.8 Other cerebral infarction
**MACE**	I639	I63.9 Cerebral infarction, unspecified
**MACE**	I64	I64 Stroke, not specified as haemorrhage or infarction
**MACE**	I650	I65.0 Occlusion and stenosis of vertebral artery
**MACE**	I651	I65.1 Occlusion and stenosis of basilar artery
**MACE**	I652	I65.2 Occlusion and stenosis of carotid artery
**MACE**	I653	I65.3 Occlusion and stenosis of multiple and bilateral precerebral arteries
**MACE**	I658	I65.8 Occlusion and stenosis of other precerebral artery
**MACE**	I659	I65.9 Occlusion and stenosis of unspecified precerebral artery
**MACE**	I660	I66.0 Occlusion and stenosis of middle cerebral artery
**MACE**	I661	I66.1 Occlusion and stenosis of anterior cerebral artery
**MACE**	I662	I66.2 Occlusion and stenosis of posterior cerebral artery
**MACE**	I663	I66.3 Occlusion and stenosis of cerebellar arteries
**MACE**	I664	I66.4 Occlusion and stenosis of multiple and bilateral cerebral arteries
**MACE**	I668	I66.8 Occlusion and stenosis of other cerebral artery
**MACE**	I669	I66.9 Occlusion and stenosis of unspecified cerebral artery
**MACE**	I693	I69.3 Sequelae of cerebral infarction
**HF**	I110	I11.0 Hypertensive heart disease with (congestive) heart failure
**HF**	I130	I13.0 Hypertensive heart and renal disease with (congestive) heart failure
**HF**	I132	I13.2 Hypertensive heart and renal disease with both (congestive) heart failure and renal failure
**HF**	I500	I50.0 Congestive heart failure
**HF**	I501	I50.1 Left ventricular failure
**HF**	I509	I50.9 Heart failure, unspecified
**HF**	J81	J81 Pulmonary oedema

MACE, major adverse cardiovascular events; HF, heart failure

Table S2A. Demographics and main results (n=44,957)

	Mean (SD) or n(%))	Median [min, max]
Age (years)	64.1 (7.7)	65.0 [44.0, 82.0]
Sex		
Male	21631 (48.1%)	
Female	23326 (51.9%)	
Self-reported ethnicity		
White	43503 (96.8%)	
Asian	484 (1.1%)	
Chinese	131 (0.3%)	
Black	293 (0.7%)	
Mixed	216 (0.5%)	
Other	235 (0.5%)	
Height (m)	1.7 (0.1)	1.7 [1.3, 2.0]
Weight (kg)	75.9 (15.1)	74.5 [34.3, 185.0]
Body mass index (kg/m^2)	26.5 (4.4)	25.8 [14.1, 69.6]
Body surface area (m^2)	1.9 (0.2)	1.9 [1.2, 2.9]
Systolic blood pressure (mmHg)	139.1 (18.7)	137.5 [61.0, 240.5]
Diastolic blood pressure (mmHg)	77.8 (10.5)	77.5 [40.0, 169.0]
Heart rate (bpm)	68.0 (11.5)	67.0 [45.0, 156.5]
Ever smoked		
No	20640 (45.9%)	
Yes	23854 (53.1%)	
Physical activity (Total MET minutes per week)	2750.2 (2439.3)	2039.5 [0.0, 19278.0]
LV end-diastolic wall thickness (mm)	7.6 (1.1)	7.5 [4.6, 18.4]
LV end-diastolic diameter (mm)	52.0 (4.6)	51.8 [33.8, 80.8]
LV end-diastolic diameter indexed to BSA (mm/m^2)	28.1 (2.8)	28.1 [17.1, 45.5]
LV end-diastolic diameter indexed to height$^{2.7}$ (mm/m$^{2.7}$)	12.7 (1.6)	12.6 [7.6, 23.1]
LV end-diastolic volume (ml)	147.1 (33.8)	142.8 [43.1, 453.1]
LV end-diastolic volume indexed to BSA (ml/m^2)	78.8 (14.1)	77.4 [29.3, 238.8]
LV end-diastolic volume indexed to height$^{2.7}$ (ml/m$^{2.7}$)	35.4 (6.2)	34.8 [15.1, 101.6]
LV mass (g)	86.0 (22.4)	82.7 [28.5, 287.5]
LV mass indexed to BSA (g/m^2)	45.8 (8.7)	44.7 [17.4, 144.9]
LV mass indexed to height$^{2.7}$ (g/m$^{2.7}$)	20.6 (4.0)	20.1 [8.0, 62.6]
LV ejection fraction (%)	59.5 (6.2)	59.7 [11.7, 82.4]
LV contraction fraction (SV/LVMV)	109.0 (20.0)	108.2 [19.7, 277.0]
LV global function index	33.9 (4.5)	33.9 [6.1, 51.9]
LV Lamé’s wall stress (Pa)	28078.7 (4578.0)	27835.6 [8858.6, 53223.4]
Pressure-strain product (mmHg%)	2511.8 (415.8)	2483.2 [1066.0, 4633.4]
Stroke work (mmHgml)	12133.8 (3308.1)	11723.7 [2006.0, 40981.3]
Stroke work indexed to BSA (mmHgml/m^2)	6498.5 (1519.1)	6333.9 [987.6, 18908.3]
Stroke work indexed to height$^{2.7}$ (mmHgml/m$^{2.7}$)	2925.6 (710.8)	2846.2 [420.3, 9221.3]
Stroke work indexed to LV mass (mmHgml/g)	143.4 (28.7)	141.2 [25.2, 345.7]
Global longitudinal strain (GLS) (-%)	18.1 (2.0)	18.1 [12.4, 23.7]
GLASE (mJ)	204.8 (57.3)	197.6 [47.8, 733.2]
GLASE indexed to BSA (mJ/m^2)	109.9 (26.9)	107.1 [32.5, 336.9]
GLASE indexed to height$^{2.7}$ (mJ/m$^{2.7}$)	49.4 (12.5)	48.1 [15.5, 161.8]
GLASED (kJ/m^3)	2.6 (0.6)	2.5 [0.8, 5.8]

Table S2B. Baseline characteristics stratified by cardiovascular disease status

	Pre-existing cardiovascular disease		
	**No**	**Yes**	***P* value**
	(N=41091)	(N=3866)	
**Age (years)**			
**Mean (SD)**	63.7 (7.69)	68.4 (6.85)	<0.001
**Median [Min, Max]**	64.0 [44.0, 82.0]	69.0 [46.0, 82.0]	
**Sex**			
**Male**	18945 (46.1%)	2686 (69.5%)	<0.001
**Female**	22146 (53.9%)	1180 (30.5%)	
**Self-reported ethnicity**			
**White**	39749 (96.7%)	3754 (97.1%)	0.00173
**Asian**	428 (1.0%)	56 (1.4%)	
**Chinese**	126 (0.3%)	5 (0.1%)	
**Black**	281 (0.7%)	12 (0.3%)	
**Mixed**	203 (0.5%)	13 (0.3%)	
**Other**	218 (0.5%)	17 (0.4%)	
**Height (m)**			
**Mean (SD)**	1.69 (0.0926)	1.71 (0.0898)	<0.001
**Median [Min, Max]**	1.69 [1.34, 2.04]	1.72 [1.44, 1.97]	
**Weight (kg)**			
**Mean (SD)**	75.5 (15.0)	80.5 (15.0)	<0.001
**Median [Min, Max]**	74.0 [34.3, 185]	79.2 [39.6, 170]	
**Body mass index (kg/m²)**			
**Mean (SD)**	26.4 (4.36)	27.5 (4.45)	<0.001
**Median [Min, Max]**	25.8 [14.1, 69.6]	26.8 [14.5, 55.1]	
**Body surface area (m²)**			
**Mean (SD)**	1.85 (0.206)	1.92 (0.199)	<0.001
**Median [Min, Max]**	1.84 [1.21, 2.86]	1.92 [1.36, 2.81]	
**Systolic blood pressure (mmHg)**			
**Mean (SD)**	139 (18.6)	141 (18.7)	<0.001
**Median [Min, Max]**	138 [61.0, 241]	140 [62.0, 215]	
**Diastolic blood pressure (mmHg)**			
**Mean (SD)**	77.9 (10.5)	76.6 (10.2)	<0.001
**Median [Min, Max]**	77.5 [40.0, 169]	76.5 [41.0, 115]	
**Heart rate (bpm)**			
**Mean (SD)**	68.3 (11.4)	65.4 (11.8)	<0.001
**Median [Min, Max]**	67.0 [45.0, 157]	64.0 [45.0, 125]	
**Ever smoked**			
**No**	19095 (46.5%)	1545 (40.0%)	<0.001
**Yes**	21590 (52.5%)	2264 (58.6%)	
**Physical activity (Total MET minutes per week)**			
**Mean (SD)**	2760 (2440)	2640 (2430)	0.00369
**Median [Min, Max]**	2060 [0, 19300]	1920 [0, 17900]	
**Hypertension**			
**No**	29246 (71.2%)	1178 (30.5%)	<0.001
**Yes**	11845 (28.8%)	2688 (69.5%)	
**Diabetes mellitus**			
**No**	38924 (94.7%)	3336 (86.3%)	<0.001
**Yes**	2167 (5.3%)	530 (13.7%)	
**Hyperlipidaemia**			
**No**	28091 (68.4%)	854 (22.1%)	<0.001
**Yes**	13000 (31.6%)	3012 (77.9%)	
**LV end-diastolic wall thickness (mm)**			
**Mean (SD)**	7.52 (1.11)	8.11 (1.19)	<0.001
**Median [Min, Max]**	7.42 [4.64, 14.7]	8.04 [5.18, 18.4]	
**LV end-diastolic diameter (mm)**			
**Mean (SD)**	51.9 (4.48)	53.6 (5.11)	<0.001
**Median [Min, Max]**	51.6 [33.8, 78.4]	53.3 [38.9, 80.8]	
**LV end-diastolic diameter indexed to BSA (mm/m²)**			
**Mean (SD)**	28.2 (2.74)	28.0 (2.92)	0.00613
**Median [Min, Max]**	28.1 [17.8, 45.5]	27.9 [17.1, 42.3]	

**LV end-diastolic diameter indexed to height$^{2.7}$ (mm/m$^{2.7}$)**			
**Mean (SD)**	12.7 (1.60)	12.7 (1.70)	0.797
**Median [Min, Max]**	12.6 [7.55, 23.1]	12.5 [7.99, 20.5]	
**LV end-diastolic volume (ml)**			
**Mean (SD)**	146 (33.2)	157 (38.1)	<0.001
**Median [Min, Max]**	142 [43.1, 438]	153 [63.5, 453]	
**LV end-diastolic volume indexed to BSA (ml/m^2)**			
**Mean (SD)**	78.5 (13.8)	81.6 (16.9)	<0.001
**Median [Min, Max]**	77.2 [29.3, 219]	79.9 [40.4, 239]	
**LV end-diastolic volume indexed to height$^{2.7}$ (ml/m$^{2.7}$)**			
**Mean (SD)**	35.2 (6.00)	36.8 (7.55)	<0.001
**Median [Min, Max]**	34.7 [15.1, 96.6]	36.1 [19.4, 102]	
**LV mass (g)**			
**Mean (SD)**	85.1 (22.0)	94.9 (23.8)	<0.001
**Median [Min, Max]**	81.6 [28.5, 248]	93.1 [39.5, 288]	
**LV mass indexed to BSA (g/m^2)**			
**Mean (SD)**	45.5 (8.50)	49.0 (9.72)	<0.001
**Median [Min, Max]**	44.3 [17.4, 145]	48.1 [23.4, 130]	
**LV mass indexed to height$^{2.7}$ (g/m$^{2.7}$)**			
**Mean (SD)**	20.4 (3.88)	22.2 (4.56)	<0.001
**Median [Min, Max]**	20.0 [8.01, 62.6]	21.6 [10.8, 62.6]	
**LV ejection fraction (%)**			
**Mean (SD)**	59.7 (5.97)	57.6 (7.83)	<0.001
**Median [Min, Max]**	59.8 [13.4, 81.1]	58.4 [11.7, 82.4]	
**LV contraction fraction (SV/LVMV)**			
**Mean (SD)**	110 (19.8)	102 (20.4)	<0.001
**Median [Min, Max]**	109 [19.7, 277]	101 [24.5, 175]	
**LV global functional index**			
**Mean (SD)**	34.1 (4.35)	32.4 (5.22)	<0.001
**Median [Min, Max]**	34.0 [6.33, 52.0]	32.7 [6.08, 48.1]	
**LV Lamé's wall stress (Pa)**			
**Mean (SD)**	28200 (4540)	27200 (4930)	<0.001
**Median [Min, Max]**	27900 [11100, 53200]	27100 [8860, 46100]	
**Pressure-strain product (mmHg%)**			
**Mean (SD)**	2520 (413)	2470 (440)	<0.001
**Median [Min, Max]**	2480 [1070, 4630]	2460 [1130, 4060]	
**Stroke work (mmHgml)**			
**Mean (SD)**	12100 (3290)	12600 (3500)	<0.001
**Median [Min, Max]**	11700 [2010, 37100]	12400 [3190, 41000]	
**Stroke work indexed to BSA (mmHgml/m^2)**			
**Mean (SD)**	6490 (1500)	6580 (1660)	0.00247
**Median [Min, Max]**	6320 [988, 18900]	6470 [1810, 18800]	
**Stroke work indexed to height$^{2.7}$ (mmHgml/m$^{2.7}$)**			
**Mean (SD)**	2920 (704)	2970 (781)	<0.001
**Median [Min, Max]**	2840 [420, 9220]	2910 [775, 9040]	
**Stroke work indexed to LV mass (mmHgml/g)**			
**Mean (SD)**	144 (28.4)	136 (30.5)	<0.001
**Median [Min, Max]**	142 [25.2, 346]	134 [30.7, 268]	
**Global longitudinal strain (GLS) (%)**			
**Mean (SD)**	18.1 (2.02)	17.6 (2.22)	<0.001
**Median [Min, Max]**	18.2 [12.4, 23.7]	17.6 [12.4, 23.6]	
**GLASE (mJ)**			
**Mean (SD)**	204 (57.0)	212 (60.5)	<0.001
**Median [Min, Max]**	197 [47.8, 601]	206 [53.4, 733]	
**GLASE indexed to BSA (mJ/m^2)**			
**Mean (SD)**	110 (26.7)	110 (29.0)	0.212
**Median [Min, Max]**	107 [32.5, 305]	108 [34.0, 337]	
**GLASE indexed to height$^{2.7}$ (mJ/m$^{2.7}$)**			
**Mean (SD)**	49.4 (12.4)	49.9 (13.5)	0.0296
**Median [Min, Max]**	48.1 [15.5, 130]	48.6 [16.9, 162]	
**GLASED (kJ/m^3)**			
**Mean (SD)**	2.57 (0.550)	2.42 (0.576)	<0.001
**Median [Min, Max]**	2.54 [0.838, 5.79]	2.39 [0.971, 4.67]	

Cardiovascular disease status was ascertained from self-reported medical history taken at the time of the visit to the imaging centre and included angina, heart attack/myocardial infarction, heart failure/pulmonary oedema, arrhythmias, stroke, peripheral vascular disease, valvular heart disease, cardiomyopathy and pericardial disease and prevalent MACE and heart failure identified from hospital episode statistics (Table S1).

Table S3A. Cox regression analysis of potential prognostic markers for all-cause mortality (Holm–Bonferroni corrected *P*<0.05 in bold)

	Model 1		Model 2		
**LV marker**	**Hazard ratio (95% CI)**	***P* value**	**Hazard ratio (95% CI)**	***P* value**	**C-statistic**
**↑ LV end-diastolic diameter**	1.02 (1.00 to 1.04)	0.109	1.02 (1.00 to 1.05)	0.051	0.787
**↓ LV end-diastolic diameter indexed to BSA**	1.00 (0.97 to 1.04)	0.818	1.05 (1.00 to 1.09)	0.044	0.787
**↑ LV end-diastolic diameter indexed to height$^{2.7}$**	1.02 (0.95 to 1.10)	0.552	1.04 (0.97 to 1.13)	0.249	0.789
**↑ LV end-diastolic volume**	1.00 (1.00 to 1.01)	**0.033**	1.00 (1.00 to 1.01)	0.030	0.789
**↑ LV end-diastolic volume indexed to BSA**	1.00 (1.00 to 1.01)	0.165	1.01 (1.00 to 1.02)	0.016	0.790
**↑ LV end-diastolic volume indexed to height$^{2.7}$**	1.01 (1.00 to 1.03)	0.044	1.02 (1.00 to 1.03)	0.021	0.790
**↑ LV mass**	1.01 (1.01 to 1.02)	**<0.0001**	1.01 (1.00 to 1.02)	**0.002**	0.788
**↑ LV mass indexed to BSA**	1.02 (1.01 to 1.03)	**0.0001**	1.02 (1.01 to 1.03)	**0.001**	0.788
**↑ LV mass indexed to height$^{2.7}$**	1.05 (1.02 to 1.07)	**<0.0001**	1.04 (1.02 to 1.07)	**0.002**	0.787
**↓ LV ejection fraction**	1.03 (1.01 to 1.04)	**0.0001**	1.03 (1.01 to 1.04)	**0.0001**	0.787
**↓ LV contraction fraction (SV/LVMV)**	1.01 (1.01 to 1.02)	**<0.0001**	1.01 (1.00 to 1.02)	**0.001**	0.793
**↓ LV global function index**	1.05 (1.03 to 1.07)	**<0.0001**	1.05 (1.02 to 1.07)	**<0.0001**	0.790
**↓ LV Lamé's wall stress**	1.00 (1.00 to 1.00)	0.059	1.00 (1.00 to 1.00)	0.476	0.786
**↓ Pressure-strain product**	1.00 (1.00 to 1.00)	0.118	1.00 (1.00 to 1.00)	0.087	0.788
**↓ Stroke work**	1.00 (1.00 to 1.00)	0.658	1.00 (1.00 to 1.00)	0.439	0.788
**↓ Stroke work indexed to BSA**	1.00 (1.00 to 1.00)	0.240	1.00 (1.00 to 1.00)	0.408	0.789
**↓ Stroke work indexed to height$^{2.7}$**	1.00 (1.00 to 1.00)	0.460	1.00 (1.00 to 1.00)	0.361	0.790
**↓ Stroke work indexed to LV mass**	1.01 (1.00 to 1.01)	**0.0002**	1.01 (1.00 to 1.01)	0.005	0.791
**↓ Global longitudinal strain**	1.09 (1.04 to 1.15)	**0.0007**	1.09 (1.03 to 1.15)	**0.001**	0.792
**↓ GLASE**	1.00 (1.00 to 1.00)	0.480	1.00 (1.00 to 1.00)	0.436	0.787
**↓ GLASE indexed to BSA**	1.00 (1.00 to 1.01)	0.169	1.00 (1.00 to 1.01)	0.335	0.788
**↓ GLASE indexed to height$^{2.7}$**	1.00 (1.00 to 1.01)	0.269	1.00 (1.00 to 1.01)	0.352	0.790
**↓ GLASED**	1.38 (1.13 to 1.68)	**0.001**	1.28 (1.04 to 1.57)	0.019	0.787

Model 1 was adjusted for age and sex, and Model 2 was adjusted for age, sex and cardiovascular risk factors (body mass index, hypertension, diabetes mellitus, dyslipidaemia, smoking history, regular alcohol intake, physical activity).

Table S3B. Cox regression analysis of potential prognostic markers for major adverse cardiovascular events (Holm–Bonferroni corrected *P*<0.05 in bold)

	Model 1		Model 2		
**LV marker**	**Hazard ratio (95% CI)**	***P* value**	**Hazard ratio (95% CI)**	***P* value**	**C-statistic**
**↑ LV end-diastolic diameter**	1.03 (1.01 to 1.05)	**0.0004**	1.03 (1.02 to 1.05)	**<0.0001**	0.718
**↓ LV end-diastolic diameter indexed to BSA**	1.00 (0.97 to 1.03)	0.999	1.06 (1.03 to 1.10)	**<0.0001**	0.715
**↑ LV end-diastolic diameter indexed to height$^{2.7}$**	1.11 (1.05 to 1.16)	**<0.0001**	1.10 (1.04 to 1.16)	**0.0003**	0.714
**↑ LV end-diastolic volume**	1.00 (1.00 to 1.01)	**<0.0001**	1.01 (1.00 to 1.01)	**<0.0001**	0.716
**↑ LV end-diastolic volume indexed to BSA**	1.01 (1.00 to 1.01)	**0.003**	1.01 (1.01 to 1.02)	**<0.0001**	0.714
**↑ LV end-diastolic volume indexed to height$^{2.7}$**	1.03 (1.02 to 1.04)	**<0.0001**	1.03 (1.02 to 1.04)	**<0.0001**	0.714
**↑ LV mass**	1.02 (1.02 to 1.02)	**<0.0001**	1.02 (1.02 to 1.02)	**<0.0001**	0.732
**↑ LV mass indexed to BSA**	1.05 (1.04 to 1.06)	**<0.0001**	1.05 (1.04 to 1.05)	**<0.0001**	0.730
**↑ LV mass indexed to height$^{2.7}$**	1.10 (1.09 to 1.12)	**<0.0001**	1.10 (1.08 to 1.12)	**<0.0001**	0.727
**↓ LV ejection fraction**	1.03 (1.02 to 1.04)	**<0.0001**	1.03 (1.02 to 1.04)	**<0.0001**	0.714
**↓ LV contraction fraction (SV/LVMV)**	1.02 (1.02 to 1.03)	**<0.0001**	1.02 (1.01 to 1.02)	**<0.0001**	0.729
**↓ LV global function index**	1.07 (1.05 to 1.08)	**<0.0001**	1.06 (1.04 to 1.08)	**<0.0001**	0.719
**↓ LV Lamé's wall stress**	1.00 (1.00 to 1.00)	**0.004**	1.00 (1.00 to 1.00)	0.259	0.715
**↑ Pressure-strain product**	1.00 (1.00 to 1.00)	0.664	1.00 (1.00 to 1.00)	0.825	0.715
**↑ Stroke work**	1.00 (1.00 to 1.00)	**0.0008**	1.00 (1.00 to 1.00)	**0.007**	0.714
**↑ Stroke work indexed to BSA**	1.00 (1.00 to 1.00)	0.019	1.00 (1.00 to 1.00)	**0.004**	0.713
**↑ Stroke work indexed to height$^{2.7}$**	1.00 (1.00 to 1.00)	**<0.0001**	1.00 (1.00 to 1.00)	**0.003**	0.712
**↓ Stroke work indexed to LV mass**	1.01 (1.00 to 1.01)	**<0.0001**	1.01 (1.00 to 1.01)	**<0.0001**	0.720
**↓ Global longitudinal strain**	1.12 (1.08 to 1.16)	**<0.0001**	1.10 (1.06 to 1.14)	**<0.0001**	0.712
**↑ GLASE**	1.00 (1.00 to 1.00)	0.021	1.00 (1.00 to 1.00)	0.028	0.717
**↑ GLASE indexed to BSA**	1.00 (1.00 to 1.00)	0.192	1.00 (1.00 to 1.01)	0.022	0.716
**↑ GLASE indexed to height$^{2.7}$**	1.01 (1.00 to 1.01)	**0.004**	1.01 (1.00 to 1.01)	0.016	0.716
**↓ GLASED**	1.39 (1.21 to 1.61)	**<0.0001**	1.25 (1.08 to 1.44)	**0.003**	0.717

Model 1 was adjusted for age and sex, and Model 2 was adjusted for age, sex and cardiovascular risk factors (body mass index, hypertension, diabetes mellitus, dyslipidaemia, smoking history, regular alcohol intake, physical activity).

Table S3C. Cox regression analysis of potential prognostic markers for heart failure (Holm–Bonferroni corrected *P*<0.05 in bold)

	Model 1		Model 2		
**LV marker**	**Hazard ratio (95% CI)**	***P* value**	**Hazard ratio (95% CI)**	***P* value**	**C-statistic**
**↑ LV end-diastolic diameter**	1.16 (1.14 to 1.19)	**<0.0001**	1.16 (1.13 to 1.19)	**<0.0001**	0.779
**↑ LV end-diastolic diameter indexed to BSA**	1.15 (1.10 to 1.20)	**<0.0001**	1.29 (1.23 to 1.35)	**<0.0001**	0.720
**↑ LV end-diastolic diameter indexed to height$^{2.7}$**	1.45 (1.33 to 1.57)	**<0.0001**	1.40 (1.28 to 1.53)	**<0.0001**	0.699
**↑ LV end-diastolic volume**	1.02 (1.01 to 1.02)	**<0.0001**	1.02 (1.01 to 1.02)	**<0.0001**	0.760
**↑ LV end-diastolic volume indexed to BSA**	1.03 (1.03 to 1.04)	**<0.0001**	1.04 (1.03 to 1.04)	**<0.0001**	0.748
**↑ LV end-diastolic volume indexed to height$^{2.7}$**	1.08 (1.07 to 1.10)	**<0.0001**	1.08 (1.07 to 1.09)	**<0.0001**	0.734
**↑ LV mass**	1.03 (1.03 to 1.03)	**<0.0001**	1.03 (1.02 to 1.03)	**<0.0001**	0.743
**↑ LV mass indexed to BSA**	1.06 (1.05 to 1.07)	**<0.0001**	1.06 (1.05 to 1.07)	**<0.0001**	0.730
**↑ LV mass indexed to height$^{2.7}$**	1.14 (1.13 to 1.16)	**<0.0001**	1.14 (1.12 to 1.16)	**<0.0001**	0.713
**↓ LV ejection fraction**	1.11 (1.10 to 1.13)	**<0.0001**	1.11 (1.09 to 1.12)	**<0.0001**	0.786
**↓ LV contraction fraction (SV/LVMV)**	1.04 (1.03 to 1.05)	**<0.0001**	1.03 (1.02 to 1.04)	**<0.0001**	0.735
**↓ LV global function index**	1.19 (1.16 to 1.21)	**<0.0001**	1.17 (1.15 to 1.20)	**<0.0001**	0.773
**↑ LV Lamé's wall stress**	1.00 (1.00 to 1.00)	0.322	1.00 (1.00 to 1.00)	0.026	0.731
**↓ Pressure-strain product**	1.00 (1.00 to 1.00)	**0.002**	1.00 (1.00 to 1.00)	**0.0002**	0.748
**↑ Stroke work**	1.00 (1.00 to 1.00)	0.043	1.00 (1.00 to 1.00)	0.392	0.707
**↑ Stroke work indexed to BSA**	1.00 (1.00 to 1.00)	0.555	1.00 (1.00 to 1.00)	0.438	0.702
**↑ Stroke work indexed to height$^{2.7}$**	1.00 (1.00 to 1.00)	0.017	1.00 (1.00 to 1.00)	0.459	0.699
**↓ Stroke work indexed to LV mass**	1.02 (1.01 to 1.02)	**<0.0001**	1.02 (1.01 to 1.02)	**<0.0001**	0.728
**↓ Global longitudinal strain**	1.30 (1.21 to 1.40)	**<0.0001**	1.28 (1.19 to 1.38)	**<0.0001**	0.793
**↑ GLASE**	1.00 (1.00 to 1.01)	**0.0007**	1.00 (1.00 to 1.01)	**0.005**	0.708
**↑ GLASE indexed to BSA**	1.01 (1.00 to 1.01)	**0.005**	1.01 (1.00 to 1.01)	**0.003**	0.703
**↑ GLASE indexed to height$^{2.7}$**	1.02 (1.01 to 1.03)	**<0.0001**	1.02 (1.01 to 1.03)	**0.003**	0.700
**↓ GLASED**	1.41 (1.06 to 1.88)	0.019	1.24 (0.93 to 1.67)	0.147	0.726

Model 1 was adjusted for age and sex, and Model 2 was adjusted for age, sex and cardiovascular risk factors (body mass index, hypertension, diabetes mellitus, dyslipidaemia, smoking history, regular alcohol intake, physical activity).

Table S4. Comparison of hazard ratios according to GLASED vs other potential prognostic markers

Model	Outcome	Marker 1 (P1)	Marker 2 (P2)	P1 Hazard ratio (95% CI)	P1 *P* value	P2 Hazard ratio (95% CI)	P2 *P* value	Comparison *P* value* (P1 vs P2 HR)
**Model 1**	All-cause mortality	↓ GLASED	↓ Global longitudinal strain	1.38 (1.13 to 1.68)	0.001	1.09 (1.04 to 1.15)	7e-04	<0.0001
		↓ GLASED	↓ LV global functional index	1.38 (1.13 to 1.68)	0.001	1.05 (1.03 to 1.07)	<0.0001	<0.0001
		↓ GLASED	↑ LV mass indexed to height$^{2.7}$	1.38 (1.13 to 1.68)	0.001	1.05 (1.02 to 1.07)	<0.0001	<0.0001
		↓ GLASED	↓ LV ejection fraction	1.38 (1.13 to 1.68)	0.001	1.03 (1.01 to 1.04)	1e-04	<0.0001
		↓ GLASED	↑ LV mass indexed to BSA	1.38 (1.13 to 1.68)	0.001	1.02 (1.01 to 1.03)	1e-04	<0.0001
		↓ GLASED	↑ LV end-diastolic diameter	1.38 (1.13 to 1.68)	0.001	1.02 (1.00 to 1.04)	0.109	<0.0001
		↓ GLASED	↑ LV end-diastolic diameter indexed to height$^{2.7}$	1.38 (1.13 to 1.68)	0.001	1.02 (0.95 to 1.10)	0.552	<0.0001
		↓ GLASED	↑ LV mass	1.38 (1.13 to 1.68)	0.001	1.01 (1.01 to 1.02)	<0.0001	<0.0001
		↓ GLASED	↓ LV contraction fraction	1.38 (1.13 to 1.68)	0.001	1.01 (1.01 to 1.02)	<0.0001	<0.0001
		↓ GLASED	↑ LV end-diastolic volume indexed to height$^{2.7}$	1.38 (1.13 to 1.68)	0.001	1.01 (1.00 to 1.03)	0.044	<0.0001
		↓ GLASED	↓ Stroke work indexed to LV mass	1.38 (1.13 to 1.68)	0.001	1.01 (1.00 to 1.01)	2e-04	<0.0001
		↓ GLASED	↑ LV end-diastolic volume	1.38 (1.13 to 1.68)	0.001	1.00 (1.00 to 1.01)	0.033	<0.0001
		↓ GLASED	↑ LV end-diastolic volume indexed to BSA	1.38 (1.13 to 1.68)	0.001	1.00 (1.00 to 1.01)	0.165	<0.0001
		↓ GLASED	↓ GLASE indexed to BSA	1.38 (1.13 to 1.68)	0.001	1.00 (1.00 to 1.01)	0.169	<0.0001
		↓ GLASED	↓ GLASE indexed to height$^{2.7}$	1.38 (1.13 to 1.68)	0.001	1.00 (1.00 to 1.01)	0.269	<0.0001
		↓ GLASED	↓ LV Lamé's wall stress	1.38 (1.13 to 1.68)	0.001	1.00 (1.00 to 1.00)	0.059	<0.0001
		↓ GLASED	↓ Pressure-strain product	1.38 (1.13 to 1.68)	0.001	1.00 (1.00 to 1.00)	0.118	<0.0001
		↓ GLASED	↓ Stroke work	1.38 (1.13 to 1.68)	0.001	1.00 (1.00 to 1.00)	0.658	<0.0001
		↓ GLASED	↓ Stroke work indexed to BSA	1.38 (1.13 to 1.68)	0.001	1.00 (1.00 to 1.00)	0.240	<0.0001
		↓ GLASED	↓ Stroke work indexed to height$^{2.7}$	1.38 (1.13 to 1.68)	0.001	1.00 (1.00 to 1.00)	0.460	<0.0001
		↓ GLASED	↓ GLASE	1.38 (1.13 to 1.68)	0.001	1.00 (1.00 to 1.00)	0.480	<0.0001
		↓ GLASED	↓ LV end-diastolic diameter indexed to BSA	1.38 (1.13 to 1.68)	0.001	1.00 (0.97 to 1.04)	0.818	<0.0001
**Model 2**		↓ GLASED	↓ Global longitudinal strain	1.28 (1.04 to 1.57)	0.019	1.09 (1.03 to 1.15)	0.001	<0.0001
		↓ GLASED	↓ LV global functional index	1.28 (1.04 to 1.57)	0.019	1.05 (1.02 to 1.07)	<0.0001	<0.0001

		↓ GLASED	↑ LV end-diastolic diameter indexed to BSA	1.28 (1.04 to 1.57)	0.019	1.05 (1.00 to 1.09)	0.044	<0.0001
		↓ GLASED	↑ LV mass indexed to height$^{2.7}$	1.28 (1.04 to 1.57)	0.019	1.04 (1.02 to 1.07)	0.002	<0.0001
		↓ GLASED	↑ LV end-diastolic diameter indexed to height$^{2.7}$	1.28 (1.04 to 1.57)	0.019	1.04 (0.97 to 1.13)	0.249	<0.0001
		↓ GLASED	↓ LV ejection fraction	1.28 (1.04 to 1.57)	0.019	1.03 (1.01 to 1.04)	1e-04	<0.0001
		↓ GLASED	↑ LV mass indexed to BSA	1.28 (1.04 to 1.57)	0.019	1.02 (1.01 to 1.03)	0.001	<0.0001
		↓ GLASED	↑ LV end-diastolic diameter	1.28 (1.04 to 1.57)	0.019	1.02 (1.00 to 1.05)	0.051	<0.0001
		↓ GLASED	↑ LV end-diastolic volume indexed to height$^{2.7}$	1.28 (1.04 to 1.57)	0.019	1.02 (1.00 to 1.03)	0.021	<0.0001
		↓ GLASED	↑ LV end-diastolic volume indexed to BSA	1.28 (1.04 to 1.57)	0.019	1.01 (1.00 to 1.02)	0.016	<0.0001
		↓ GLASED	↑ LV mass	1.28 (1.04 to 1.57)	0.019	1.01 (1.00 to 1.02)	0.002	<0.0001
		↓ GLASED	↓ LV contraction fraction	1.28 (1.04 to 1.57)	0.019	1.01 (1.00 to 1.02)	0.001	<0.0001
		↓ GLASED	↓ Stroke work indexed to LV mass	1.28 (1.04 to 1.57)	0.019	1.01 (1.00 to 1.01)	0.005	<0.0001
		↓ GLASED	↑ LV end-diastolic volume	1.28 (1.04 to 1.57)	0.019	1.00 (1.00 to 1.01)	0.030	<0.0001
		↓ GLASED	↓ GLASE indexed to BSA	1.28 (1.04 to 1.57)	0.019	1.00 (1.00 to 1.01)	0.335	<0.0001
		↓ GLASED	↓ GLASE indexed to height$^{2.7}$	1.28 (1.04 to 1.57)	0.019	1.00 (1.00 to 1.01)	0.352	<0.0001
		↓ GLASED	↓ LV Lamé's wall stress	1.28 (1.04 to 1.57)	0.019	1.00 (1.00 to 1.00)	0.476	<0.0001
		↓ GLASED	↓ Pressure-strain product	1.28 (1.04 to 1.57)	0.019	1.00 (1.00 to 1.00)	0.087	<0.0001
		↓ GLASED	↓ Stroke work	1.28 (1.04 to 1.57)	0.019	1.00 (1.00 to 1.00)	0.439	<0.0001
		↓ GLASED	↓ Stroke work indexed to BSA	1.28 (1.04 to 1.57)	0.019	1.00 (1.00 to 1.00)	0.408	<0.0001
		↓ GLASED	↓ Stroke work indexed to height$^{2.7}$	1.28 (1.04 to 1.57)	0.019	1.00 (1.00 to 1.00)	0.361	<0.0001
		↓ GLASED	↓ GLASE	1.28 (1.04 to 1.57)	0.019	1.00 (1.00 to 1.00)	0.436	<0.0001
**Model 1**	MACE	↓ GLASED	↓ Global longitudinal strain	1.39 (1.21 to 1.61)	<0.0001	1.12 (1.08 to 1.16)	<0.0001	<0.0001
		↓ GLASED	↑ LV end-diastolic diameter indexed to height$^{2.7}$	1.39 (1.21 to 1.61)	<0.0001	1.11 (1.06 to 1.16)	<0.0001	<0.0001
		↓ GLASED	↑ LV mass indexed to height$^{2.7}$	1.39 (1.21 to 1.61)	<0.0001	1.10 (1.09 to 1.12)	<0.0001	<0.0001
		↓ GLASED	↓ LV global functional index	1.39 (1.21 to 1.61)	<0.0001	1.07 (1.05 to 1.09)	<0.0001	<0.0001
		↓ GLASED	↑ LV mass indexed to BSA	1.39 (1.21 to 1.61)	<0.0001	1.05 (1.04 to 1.06)	<0.0001	<0.0001
		↓ GLASED	↑ LV end-diastolic volume indexed to height$^{2.7}$	1.39 (1.21 to 1.61)	<0.0001	1.03 (1.02 to 1.04)	<0.0001	<0.0001
		↓ GLASED	↓ LV ejection fraction	1.39 (1.21 to 1.61)	<0.0001	1.03 (1.02 to 1.04)	<0.0001	<0.0001
		↓ GLASED	↑ LV end-diastolic diameter	1.39 (1.21 to 1.61)	<0.0001	1.03 (1.01 to 1.05)	4e-04	<0.0001

		↓ GLASED	↓ LV contraction fraction	1.39 (1.21 to 1.61)	<0.0001	1.02 (1.02 to 1.03)	<0.0001	<0.0001
		↓ GLASED	↑ LV mass	1.39 (1.21 to 1.61)	<0.0001	1.02 (1.02 to 1.02)	<0.0001	<0.0001
		↓ GLASED	↑ LV end-diastolic volume indexed to BSA	1.39 (1.21 to 1.61)	<0.0001	1.01 (1.00 to 1.01)	0.003	<0.0001
		↓ GLASED	↓ Stroke work indexed to LV mass	1.39 (1.21 to 1.61)	<0.0001	1.01 (1.00 to 1.01)	<0.0001	<0.0001
		↓ GLASED	↑ GLASE indexed to height$^{2.7}$	1.39 (1.21 to 1.61)	<0.0001	1.01 (1.00 to 1.01)	0.005	<0.0001
		↓ GLASED	↑ LV end-diastolic volume	1.39 (1.21 to 1.61)	<0.0001	1.00 (1.00 to 1.01)	<0.0001	<0.0001
		↓ GLASED	↓ LV Lamé's wall stress	1.39 (1.21 to 1.61)	<0.0001	1.00 (1.00 to 1.00)	0.004	<0.0001
		↓ GLASED	↑ Pressure-strain product	1.39 (1.21 to 1.61)	<0.0001	1.00 (1.00 to 1.00)	0.681	<0.0001
		↓ GLASED	↑ Stroke work	1.39 (1.21 to 1.61)	<0.0001	1.00 (1.00 to 1.00)	9e-04	<0.0001
		↓ GLASED	↑ Stroke work indexed to BSA	1.39 (1.21 to 1.61)	<0.0001	1.00 (1.00 to 1.00)	0.020	<0.0001
		↓ GLASED	↑ Stroke work indexed to height$^{2.7}$	1.39 (1.21 to 1.61)	<0.0001	1.00 (1.00 to 1.00)	<0.0001	<0.0001
		↓ GLASED	↑ GLASE	1.39 (1.21 to 1.61)	<0.0001	1.00 (1.00 to 1.00)	0.022	<0.0001
		↓ GLASED	↑ GLASE indexed to BSA	1.39 (1.21 to 1.61)	<0.0001	1.00 (1.00 to 1.00)	0.196	<0.0001
		↓ GLASED	↑ LV end-diastolic diameter indexed to BSA	1.39 (1.21 to 1.61)	<0.0001	1.00 (0.97 to 1.03)	0.994	<0.0001
**Model 2**		↓ GLASED	↑ LV mass indexed to height$^{2.7}$	1.25 (1.08 to 1.44)	0.003	1.10 (1.08 to 1.12)	<0.0001	<0.0001
		↓ GLASED	↓ Global longitudinal strain	1.25 (1.08 to 1.44)	0.003	1.10 (1.06 to 1.14)	<0.0001	<0.0001
		↓ GLASED	↑ LV end-diastolic diameter indexed to height$^{2.7}$	1.25 (1.08 to 1.44)	0.003	1.10 (1.04 to 1.16)	3e-04	<0.0001
		↓ GLASED	↓ LV global functional index	1.25 (1.08 to 1.44)	0.003	1.06 (1.04 to 1.08)	<0.0001	<0.0001
		↓ GLASED	↑ LV end-diastolic diameter indexed to BSA	1.25 (1.08 to 1.44)	0.003	1.06 (1.03 to 1.10)	<0.0001	<0.0001
		↓ GLASED	↑ LV mass indexed to BSA	1.25 (1.08 to 1.44)	0.003	1.05 (1.04 to 1.05)	<0.0001	<0.0001
		↓ GLASED	↑ LV end-diastolic diameter	1.25 (1.08 to 1.44)	0.003	1.03 (1.02 to 1.05)	<0.0001	<0.0001
		↓ GLASED	↑ LV end-diastolic volume indexed to height$^{2.7}$	1.25 (1.08 to 1.44)	0.003	1.03 (1.02 to 1.04)	<0.0001	<0.0001
		↓ GLASED	↓ LV ejection fraction	1.25 (1.08 to 1.44)	0.003	1.03 (1.02 to 1.04)	<0.0001	<0.0001
		↓ GLASED	↑ LV mass	1.25 (1.08 to 1.44)	0.003	1.02 (1.02 to 1.02)	<0.0001	<0.0001
		↓ GLASED	↓ LV contraction fraction	1.25 (1.08 to 1.44)	0.003	1.02 (1.01 to 1.02)	<0.0001	<0.0001
		↓ GLASED	↑ LV end-diastolic volume indexed to BSA	1.25 (1.08 to 1.44)	0.003	1.01 (1.01 to 1.02)	<0.0001	<0.0001
		↓ GLASED	↑ LV end-diastolic volume	1.25 (1.08 to 1.44)	0.003	1.01 (1.00 to 1.01)	<0.0001	<0.0001
		↓ GLASED	↓ Stroke work indexed to LV mass	1.25 (1.08 to 1.44)	0.003	1.01 (1.00 to 1.01)	<0.0001	<0.0001
		↓ GLASED	↑ GLASE indexed to height$^{2.7}$	1.25 (1.08 to 1.44)	0.003	1.01 (1.00 to 1.01)	0.016	<0.0001

		↓ GLASED	↑ GLASE indexed to BSA	1.25 (1.08 to 1.44)	0.003	1.00 (1.00 to 1.01)	0.023	<0.0001
		↓ GLASED	↓ LV Lamé's wall stress	1.25 (1.08 to 1.44)	0.003	1.00 (1.00 to 1.00)	0.256	<0.0001
		↓ GLASED	↓ Pressure-strain product	1.25 (1.08 to 1.44)	0.003	1.00 (1.00 to 1.00)	0.812	<0.0001
		↓ GLASED	↑ Stroke work	1.25 (1.08 to 1.44)	0.003	1.00 (1.00 to 1.00)	0.008	<0.0001
		↓ GLASED	↑ Stroke work indexed to BSA	1.25 (1.08 to 1.44)	0.003	1.00 (1.00 to 1.00)	0.004	<0.0001
		↓ GLASED	↑ Stroke work indexed to height$^{2.7}$	1.25 (1.08 to 1.44)	0.003	1.00 (1.00 to 1.00)	0.004	<0.0001
		↓ GLASED	↑ GLASE	1.25 (1.08 to 1.44)	0.003	1.00 (1.00 to 1.00)	0.028	<0.0001
**Model 1**	Heart failure	↓ GLASED	↑ LV end-diastolic diameter indexed to height2.7	1.41 (1.06 to 1.88)	0.018	1.45 (1.34 to 1.57)	<0.0001	ns
		↓ GLASED	↓ Global longitudinal strain	1.41 (1.06 to 1.88)	0.018	1.30 (1.21 to 1.40)	<0.0001	<0.0001
		↓ GLASED	↓ LV global functional index	1.41 (1.06 to 1.88)	0.018	1.19 (1.16 to 1.21)	<0.0001	<0.0001
		↓ GLASED	↑ LV end-diastolic diameter	1.41 (1.06 to 1.88)	0.018	1.17 (1.14 to 1.19)	<0.0001	<0.0001
		↓ GLASED	↑ LV end-diastolic diameter indexed to BSA	1.41 (1.06 to 1.88)	0.018	1.15 (1.10 to 1.20)	<0.0001	<0.0001
		↓ GLASED	↑ LV mass indexed to height2.7	1.41 (1.06 to 1.88)	0.018	1.14 (1.13 to 1.16)	<0.0001	<0.0001
		↓ GLASED	↓ LV ejection fraction	1.41 (1.06 to 1.88)	0.018	1.11 (1.10 to 1.13)	<0.0001	<0.0001
		↓ GLASED	↑ LV end-diastolic volume indexed to height2.7	1.41 (1.06 to 1.88)	0.018	1.09 (1.07 to 1.10)	<0.0001	<0.0001
		↓ GLASED	↑ LV mass indexed to BSA	1.41 (1.06 to 1.88)	0.018	1.06 (1.05 to 1.07)	<0.0001	<0.0001
		↓ GLASED	↓ LV contraction fraction	1.41 (1.06 to 1.88)	0.018	1.04 (1.03 to 1.05)	<0.0001	<0.0001
		↓ GLASED	↑ LV end-diastolic volume indexed to BSA	1.41 (1.06 to 1.88)	0.018	1.03 (1.03 to 1.04)	<0.0001	<0.0001
		↓ GLASED	↑ LV mass	1.41 (1.06 to 1.88)	0.018	1.03 (1.03 to 1.03)	<0.0001	<0.0001
		↓ GLASED	↑ LV end-diastolic volume	1.41 (1.06 to 1.88)	0.018	1.02 (1.02 to 1.02)	<0.0001	<0.0001
		↓ GLASED	↑ GLASE indexed to height$^{2.7}$	1.41 (1.06 to 1.88)	0.018	1.02 (1.01 to 1.03)	<0.0001	<0.0001
		↓ GLASED	↓ Stroke work indexed to LV mass	1.41 (1.06 to 1.88)	0.018	1.02 (1.01 to 1.02)	<0.0001	<0.0001
		↓ GLASED	↑ GLASE indexed to BSA	1.41 (1.06 to 1.88)	0.018	1.01 (1.00 to 1.01)	0.005	<0.0001
		↓ GLASED	↑ GLASE	1.41 (1.06 to 1.88)	0.018	1.00 (1.00 to 1.01)	7e-04	<0.0001
		↓ GLASED	↑ LV Lamé's wall stress	1.41 (1.06 to 1.88)	0.018	1.00 (1.00 to 1.00)	0.329	<0.0001
		↓ GLASED	↓ Pressure-strain product	1.41 (1.06 to 1.88)	0.018	1.00 (1.00 to 1.00)	0.002	<0.0001
		↓ GLASED	↑ Stroke work	1.41 (1.06 to 1.88)	0.018	1.00 (1.00 to 1.00)	0.045	<0.0001
		↓ GLASED	↑ Stroke work indexed to BSA	1.41 (1.06 to 1.88)	0.018	1.00 (1.00 to 1.00)	0.567	<0.0001

		↓ GLASED	↑ Stroke work indexed to height$^{2.7}$	1.41 (1.06 to 1.88)	0.018	1.00 (1.00 to 1.00)	0.017	<0.0001
**Model 2**		↓ GLASED	↑ LV end-diastolic diameter indexed to height$^{2.7}$	1.25 (0.93 to 1.67)	0.145	1.40 (1.29 to 1.53)	<0.0001	<0.0001
		↓ GLASED	↑ LV end-diastolic diameter indexed to BSA	1.25 (0.93 to 1.67)	0.145	1.29 (1.23 to 1.35)	<0.0001	<0.01
		↓ GLASED	↓ Global longitudinal strain	1.25 (0.93 to 1.67)	0.145	1.28 (1.19 to 1.38)	<0.0001	ns
		↓ GLASED	↓ LV global functional index	1.25 (0.93 to 1.67)	0.145	1.18 (1.15 to 1.20)	<0.0001	<0.0001
		↓ GLASED	↑ LV end-diastolic diameter	1.25 (0.93 to 1.67)	0.145	1.16 (1.14 to 1.19)	<0.0001	<0.0001
		↓ GLASED	↑ LV mass indexed to height$^{2.7}$	1.25 (0.93 to 1.67)	0.145	1.14 (1.12 to 1.16)	<0.0001	<0.0001
		↓ GLASED	↓ LV ejection fraction	1.25 (0.93 to 1.67)	0.145	1.11 (1.09 to 1.12)	<0.0001	<0.0001
		↓ GLASED	↑ LV end-diastolic volume indexed to height$^{2.7}$	1.25 (0.93 to 1.67)	0.145	1.08 (1.07 to 1.10)	<0.0001	<0.0001
		↓ GLASED	↑ LV mass indexed to BSA	1.25 (0.93 to 1.67)	0.145	1.06 (1.05 to 1.07)	<0.0001	<0.0001
		↓ GLASED	↑ LV end-diastolic volume indexed to BSA	1.25 (0.93 to 1.67)	0.145	1.04 (1.03 to 1.04)	<0.0001	<0.0001
		↓ GLASED	↓ LV contraction fraction	1.25 (0.93 to 1.67)	0.145	1.03 (1.02 to 1.04)	<0.0001	<0.0001
		↓ GLASED	↑ LV mass	1.25 (0.93 to 1.67)	0.145	1.03 (1.02 to 1.03)	<0.0001	<0.0001
		↓ GLASED	↑ LV end-diastolic volume	1.25 (0.93 to 1.67)	0.145	1.02 (1.02 to 1.02)	<0.0001	<0.0001
		↓ GLASED	↑ GLASE indexed to height$^{2.7}$	1.25 (0.93 to 1.67)	0.145	1.02 (1.01 to 1.03)	0.003	<0.0001
		↓ GLASED	↓ Stroke work indexed to LV mass	1.25 (0.93 to 1.67)	0.145	1.02 (1.01 to 1.02)	<0.0001	<0.0001
		↓ GLASED	↑ GLASE indexed to BSA	1.25 (0.93 to 1.67)	0.145	1.01 (1.00 to 1.01)	0.003	<0.0001
		↓ GLASED	↑ GLASE	1.25 (0.93 to 1.67)	0.145	1.00 (1.00 to 1.01)	0.005	<0.0001
		↓ GLASED	↑ LV Lamé's wall stress	1.25 (0.93 to 1.67)	0.145	1.00 (1.00 to 1.00)	0.026	<0.0001
		↓ GLASED	↓ Pressure-strain product	1.25 (0.93 to 1.67)	0.145	1.00 (1.00 to 1.00)	2e-04	<0.0001
		↓ GLASED	↑ Stroke work	1.25 (0.93 to 1.67)	0.145	1.00 (1.00 to 1.00)	0.407	<0.0001
		↓ GLASED	↑ Stroke work indexed to BSA	1.25 (0.93 to 1.67)	0.145	1.00 (1.00 to 1.00)	0.451	<0.0001
		↓ GLASED	↑ Stroke work indexed to height$^{2.7}$	1.25 (0.93 to 1.67)	0.145	1.00 (1.00 to 1.00)	0.471	<0.0001

*Holm–Bonferroni-corrected *P* value

Table S5A. Cox regression analysis of potential prognostic markers for all-cause mortality in the subgroup with a normal LVEF (>55%)

	Model 1		Model 2	
**LV marker**	**Hazard ratio (95% CI)**	***P* value**	**Hazard ratio (95% CI)**	***P* value**
**↓ LV end-diastolic diameter**	1.01 (0.98 to 1.04)	0.592	1.00 (0.97 to 1.03)	0.901
**↓ LV end-diastolic diameter indexed to BSA**	1.04 (1.00 to 1.09)	0.080	1.01 (0.96 to 1.07)	0.700
**↓ LV end-diastolic diameter indexed to height$^{2.7}$**	1.07 (0.98 to 1.17)	0.119	1.05 (0.95 to 1.15)	0.346
**↓ LV end-diastolic volume**	1.00 (1.00 to 1.01)	0.610	1.00 (1.00 to 1.01)	0.697
**↓ LV end-diastolic volume indexed to BSA**	1.01 (1.00 to 1.02)	0.176	1.00 (0.99 to 1.01)	0.550
**↓ LV end-diastolic volume indexed to height$^{2.7}$**	1.01 (0.99 to 1.03)	0.239	1.01 (0.99 to 1.03)	0.403
**↑ LV mass**	1.01 (1.00 to 1.01)	0.029	1.01 (1.00 to 1.01)	0.122
**↑ LV mass indexed to BSA**	1.01 (1.00 to 1.03)	0.104	1.01 (1.00 to 1.03)	0.159
**↑ LV mass indexed to height$^{2.7}$**	1.02 (0.99 to 1.06)	0.124	1.02 (0.98 to 1.06)	0.271
**↓ LV ejection fraction**	1.01 (0.98 to 1.04)	0.513	1.01 (0.98 to 1.04)	0.642
**↓ LV contraction fraction (SV/LVMV)**	1.01 (1.00 to 1.02)	0.001	1.01 (1.00 to 1.02)	0.032
**↓ LV global function index**	1.04 (1.01 to 1.08)	0.024	1.03 (0.99 to 1.07)	0.128
**↓ LV Lamé's wall stress**	1.00 (1.00 to 1.00)	0.052	1.00 (1.00 to 1.00)	0.258
**↓ Pressure-strain loop**	1.00 (1.00 to 1.00)	0.234	1.00 (1.00 to 1.00)	0.205
**↑ Stroke work**	1.00 (1.00 to 1.00)	0.994	1.00 (1.00 to 1.00)	0.990
**↓ Stroke work indexed to BSA**	1.00 (1.00 to 1.00)	0.479	1.00 (1.00 to 1.00)	0.738
**↓ Stroke work indexed to height$^{2.7}$**	1.00 (1.00 to 1.00)	0.528	1.00 (1.00 to 1.00)	0.600
**↓ Stroke work indexed to LV mass**	1.01 (1.00 to 1.01)	0.024	1.00 (1.00 to 1.01)	0.111
**↓ Global longitudinal strain**	1.10 (1.03 to 1.17)	0.003	1.09 (1.02 to 1.16)	0.013
**↓ GLASE**	1.00 (1.00 to 1.00)	0.348	1.00 (1.00 to 1.00)	0.413
**↓ GLASE indexed to BSA**	1.00 (1.00 to 1.01)	0.118	1.00 (1.00 to 1.01)	0.281
**↓ GLASE indexed to height$^{2.7}$**	1.01 (1.00 to 1.02)	0.151	1.01 (1.00 to 1.02)	0.256
**↓ GLASED**	1.38 (1.11 to 1.73)	0.005	1.28 (1.01 to 1.63)	0.038

Model 1 was adjusted for age and sex, and Model 2 was adjusted for age, sex and cardiovascular risk factors (body mass index, hypertension, diabetes mellitus, dyslipidaemia, smoking history, regular alcohol intake, physical activity).

Table S5B. Cox regression analysis of potential prognostic markers for major adverse cardiovascular events in the subgroup with a normal LVEF (>55%)

	Model 1		Model 2	
**LV marker**	**Hazard ratio (95% CI)**	***P* value**	**Hazard ratio (95% CI)**	***P* value**
**↑ LV end-diastolic diameter**	1.01 (0.99 to 1.03)	0.465	1.01 (0.99 to 1.04)	0.247
**↓ LV end-diastolic diameter indexed to BSA**	1.04 (1.00 to 1.07)	0.024	1.01 (0.97 to 1.05)	0.658
**↑ LV end-diastolic diameter indexed to height$^{2.7}$**	1.03 (0.97 to 1.09)	0.360	1.02 (0.95 to 1.08)	0.628
**↑ LV end-diastolic volume**	1.00 (1.00 to 1.00)	0.275	1.00 (1.00 to 1.01)	0.228
**↓ LV end-diastolic volume indexed to BSA**	1.00 (0.99 to 1.01)	0.731	1.00 (1.00 to 1.01)	0.282
**↑ LV end-diastolic volume indexed to height$^{2.7}$**	1.01 (1.00 to 1.02)	0.137	1.01 (0.99 to 1.02)	0.251
**↑ LV mass**	1.02 (1.01 to 1.02)	<0.0001	1.02 (1.01 to 1.02)	<0.0001
**↑ LV mass indexed to BSA**	1.04 (1.03 to 1.05)	<0.0001	1.04 (1.03 to 1.05)	<0.0001
**↑ LV mass indexed to height$^{2.7}$**	1.09 (1.07 to 1.11)	<0.0001	1.09 (1.06 to 1.11)	<0.0001
**↑ LV ejection fraction**	1.00 (0.98 to 1.02)	0.843	1.01 (0.99 to 1.03)	0.556
**↓ LV contraction fraction (SV/LVMV)**	1.02 (1.01 to 1.03)	<0.0001	1.02 (1.01 to 1.02)	<0.0001
**↓ LV global function index**	1.06 (1.03 to 1.09)	<0.0001	1.05 (1.02 to 1.08)	0.0003
**↓ LV Lamé's wall stress**	1.00 (1.00 to 1.00)	0.012	1.00 (1.00 to 1.00)	0.200
**↑ Pressure-strain loop**	1.00 (1.00 to 1.00)	0.179	1.00 (1.00 to 1.00)	0.383
**↑ Stroke work**	1.00 (1.00 to 1.00)	0.0001	1.00 (1.00 to 1.00)	0.002
**↑ Stroke work indexed to BSA**	1.00 (1.00 to 1.00)	0.002	1.00 (1.00 to 1.00)	0.001
**↑ Stroke work indexed to height$^{2.7}$**	1.00 (1.00 to 1.00)	<0.0001	1.00 (1.00 to 1.00)	0.001
**↓ Stroke work indexed to LV mass**	1.00 (1.00 to 1.01)	0.011	1.00 (1.00 to 1.01)	0.089
**↓ Global longitudinal strain**	1.13 (1.08 to 1.18)	<0.0001	1.11 (1.05 to 1.16)	<0.0001
**↑ GLASE**	1.00 (1.00 to 1.00)	0.116	1.00 (1.00 to 1.00)	0.174
**↑ GLASE indexed to BSA**	1.00 (1.00 to 1.00)	0.510	1.00 (1.00 to 1.01)	0.178
**↑ GLASE indexed to height$^{2.7}$**	1.01 (1.00 to 1.01)	0.060	1.00 (1.00 to 1.01)	0.160
**↓ GLASED**	1.39 (1.18 to 1.64)	<0.0001	1.25 (1.06 to 1.48)	0.009

Model 1 was adjusted for age and sex, and Model 2 was adjusted for age, sex and cardiovascular risk factors (body mass index, hypertension, diabetes mellitus, dyslipidaemia, smoking history, regular alcohol intake, physical activity).

Table S5C. Cox regression analysis of potential prognostic markers for heart failure in the subgroup with a normal LVEF (>55%)

	Model 1		Model 2	
**LV marker**	**Hazard ratio (95% CI)**	***P* value**	**Hazard ratio (95% CI)**	***P* value**
**↑ LV end-diastolic diameter**	1.10 (1.05 to 1.15)	<0.0001	1.08 (1.03 to 1.14)	0.001
**↑ LV end-diastolic diameter indexed to BSA**	1.00 (0.93 to 1.08)	0.929	1.18 (1.08 to 1.28)	0.0002
**↑ LV end-diastolic diameter indexed to height$^{2.7}$**	1.35 (1.19 to 1.53)	<0.0001	1.25 (1.09 to 1.44)	0.001
**↑ LV end-diastolic volume**	1.01 (1.01 to 1.02)	<0.0001	1.01 (1.01 to 1.02)	0.0004
**↑ LV end-diastolic volume indexed to BSA**	1.02 (1.01 to 1.04)	0.002	1.03 (1.02 to 1.04)	<0.0001
**↑ LV end-diastolic volume indexed to height$^{2.7}$**	1.08 (1.06 to 1.11)	<0.0001	1.07 (1.04 to 1.10)	<0.0001
**↑ LV mass**	1.03 (1.02 to 1.04)	<0.0001	1.02 (1.01 to 1.03)	0.0001
**↑ LV mass indexed to BSA**	1.06 (1.04 to 1.08)	<0.0001	1.05 (1.03 to 1.07)	<0.0001
**↑ LV mass indexed to height$^{2.7}$**	1.16 (1.12 to 1.20)	<0.0001	1.11 (1.06 to 1.16)	<0.0001
**↓ LV ejection fraction**	1.01 (0.96 to 1.05)	0.791	1.01 (0.96 to 1.05)	0.762
**↓ LV contraction fraction (SV/LVMV)**	1.01 (1.00 to 1.02)	0.029	1.00 (0.99 to 1.01)	0.887
**↓ LV global function index**	1.05 (0.99 to 1.11)	0.103	1.00 (0.94 to 1.06)	0.958
**↑ LV Lamé's wall stress**	1.00 (1.00 to 1.00)	0.844	1.00 (1.00 to 1.00)	0.249
**↓ Pressure-strain loop**	1.00 (1.00 to 1.00)	0.989	1.00 (1.00 to 1.00)	0.703
**↑ Stroke work**	1.00 (1.00 to 1.00)	<0.0001	1.00 (1.00 to 1.00)	0.003
**↑ Stroke work indexed to BSA**	1.00 (1.00 to 1.00)	0.002	1.00 (1.00 to 1.00)	0.0006
**↑ Stroke work indexed to height$^{2.7}$**	1.00 (1.00 to 1.00)	<0.0001	1.00 (1.00 to 1.00)	0.0005
**↓ Stroke work indexed to LV mass**	1.00 (1.00 to 1.01)	0.570	1.00 (0.99 to 1.01)	0.595
**↓ Global longitudinal strain**	1.14 (1.03 to 1.26)	0.011	1.10 (0.99 to 1.22)	0.085
**↑ GLASE**	1.00 (1.00 to 1.01)	0.010	1.00 (1.00 to 1.01)	0.064
**↑ GLASE indexed to BSA**	1.01 (1.00 to 1.01)	0.063	1.01 (1.00 to 1.01)	0.028
**↑ GLASE indexed to height$^{2.7}$**	1.02 (1.01 to 1.04)	0.0006	1.02 (1.00 to 1.03)	0.017
**↓ GLASED**	1.17 (0.81 to 1.68)	0.401	1.03 (0.71 to 1.49)	0.888

Model 1 was adjusted for age and sex, and Model 2 was adjusted for age, sex and cardiovascular risk factors (body mass index, hypertension, diabetes mellitus, dyslipidaemia, smoking history, regular alcohol intake, physical activity).

Table S6. Atrial Fibrillation and GLASED

Variable	AF -ve	AF +ve	P
N(%)	43,636 (96.1%)	1,321 (2.9%)	
GLASED (SD) (kJ/m^3)	2.56 (0.55)	2.44 (0.61)	<0.001

A total of 1321 people (2.9%) were diagnosed with atrial fibrillation (AF) at the time of CMR imaging. The presence of atrial fibrillation was associated with lower GLASED.
